# Synthesis, Characterization and Biological Evaluation of Novel Dihydropyranoindoles Improving the Anticancer Effects of HDAC Inhibitors

**DOI:** 10.3390/molecules25061377

**Published:** 2020-03-18

**Authors:** Murat Bingul, Greg M. Arndt, Glenn M. Marshall, Belamy B. Cheung, Naresh Kumar, David StC. Black

**Affiliations:** 1 School of Chemistry, UNSW Sydney, Sydney, NSW 2052, Australia; muratbingul1983@gmail.com; 2 Children’s Cancer Institute, Lowy Cancer Research Centre, UNSW Sydney, Sydney, NSW 2052, Australia; garndt@ccia.unsw.edu.au (G.M.A.); gmarshall@ccia.unsw.edu.au (G.M.M.); 3 School of Pharmacy, Dicle University, 21280 Diyarbakır, Turkey; 4 ACRF Drug Discovery Centre for Childhood Cancer, Children’s Cancer Institute, Lowy Cancer Research Centre, UNSW Sydney, Sydney, NSW 2052, Australia; 5 Kids Cancer Centre, Sydney Children’s Hospital, Randwick, NSW 2031, Australia; 6 School of Women’s and Children’s Health, UNSW Sydney, Sydney, NSW 2052, Australia

**Keywords:** dihydropyranoindole, anticancer, HDAC inhibitors, neuroblastoma, breast cancer

## Abstract

The dihydropyranoindole scaffold was identified as a promising target for improving the anti-cancer activity of HDAC inhibitors from the preliminary screening of a library of compounds. A suitable methodology has been developed for the preparation of novel dihydropyranoindoles via the Hemetsberger indole synthesis using azido-phenylacrylates, derived from the reaction of corresponding alkynyl-benzaldehydes with methyl azidoacetate, followed by thermal cyclization in high boiling solvents. Anti-cancer activity of all the newly synthesized compounds was evaluated against the SH-SY5Y and Kelly neuroblastoma cells as well as the MDA-MB-231 and MCF-7 breast adenocarcinoma cell lines. Biological studies showed that the tetracyclic systems had significant cytotoxic activity at higher concentration against the neuroblastoma cancer cells. More importantly, these systems, at the lower concentration, considerably enhanced the SAHA toxicity. In addition to that, the toxicity of designated systems on the healthy human cells was found to be significantly less than the cancer cells.

## 1. Introduction

Chemotherapy is one of the most widely used treatments for high-risk cancer patients [1,2]. A range of well-known agents, namely Doxorubicin, Cyclophosphamide, Etoposide have been used to combat various cancers in modern chemotherapeutic therapy [3,4]. However, a major problem faced in chemotherapy is resistance to commonly-used anti-cancer drugs [5]. Therefore, development of novel anti-cancer chemotherapeutic agents is of utmost importance in the area of drug discovery and development.

The histone deacetylase (HDAC) inhibitors are a class of chemotherapeutic agents that hold promise in cancer therapy [6,7]. HDAC inhibitors have been reported to suppress cell proliferation and angiogenesis, induce cell differentiation and promote apoptosis in a number of cancer cell types [8,9]. Suberoylanilide hydroxamic acid (SAHA) is the first HDAC inhibitor to obtain meet FDA approval [10], and has been considered to be a highly promising anticancer therapeutic agent due to its potent cytotoxic effect on a number of tumor cell types as well as low toxicity towards healthy normal cells [11,12,13]. However, single agent treatment with SAHA has been found to be ineffective against several cancers [14,15]. On the other hand, the combination of SAHA with other chemotherapeutic agents with different mechanisms of action has been considered to be more promising as the drug resistance caused by single agent therapies may be overcome [14,16]. Due to the significant cytotoxic effects observed during clinical trials of SAHA in combination with a variety of chemotherapeutic agents, many synthetic compounds have been produced and screened to identify molecules capable of enhancing the cytotoxic activity of SAHA, while producing fewer side effects.

In order to provide a basis for this study, a subset (10,560 compounds) of the Walter & Eliza Hall Institute (WEHI) compound library was screened to identify molecules that can act synergistically with a clinical dose of SAHA to overcome drug resistance in SAHA-resistant MDA-MB-231 breast cancer cell lines [17]. The compounds that reduced viability to <40% in the presence of SAHA but to >70% in the absence of SAHA, with a difference of at least 55% between the two conditions have been identified as hit molecules for their SAHA enhancing capability [17]. A structural analysis of the hit molecules demonstrated that the main structural feature to be the presence of 5- or 6-membered fused heterocyclic systems, most commonly containing one or more nitrogen atoms. Furthermore, these fused systems typically involved 3 or 4 conjoined rings. Due to the ongoing experiments regarding the hit molecules, the structures of these compounds are not discussed in this manuscript.

### Identification of New Target Molecules

**Figure 1:**
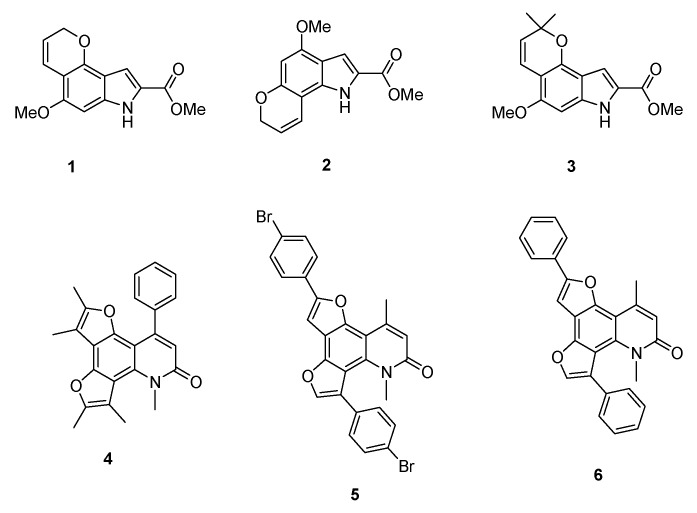
Six compounds from the NK library.

The starting point of the current work was the selection of a range of compounds exhibiting structural similarity to the hits from compound library in order to expand the structural diversity of the screening set. The six related monomeric dihydropyranoindoles **1**–**3** and dimeric furoquinolines **4**–**6** were selected in order to understand the effect of the indole and quinoline structures, as well as the fused dihydropyran and furan rings, on biological activities (Figure 1). The molecules were tested against SH-SY5Y neuroblastoma and MDA-MB-231 breast cancer cell lines, using the Alamar blue (Resazurin) assay [18] to measure the cell viability. The same screening conditions were used with WEHI screening. Briefly, the designated cells were exposed to 1 μM SAHA, 10 μM compound and the combination of SAHA and compound for 72 h.

The in vitro assays revealed that the MDA-MB-231 were the more resistant cell lines than the SH-SY5Y cell lines for the single and combination treatments of all compounds. The compounds **5** and **6**, analogues of quinolines, displayed the lowest reduction on SH-SY5Y cell viability and also no SAHA enhancement was found by the combination with SAHA (Figure S1A, see Supplementary Materials). In the case of compound **6**, the single and combination treatments showed no inhibition of MDA-MB-231 cell viability, while the 20% and 5% of inhibitions were obtained with the treatment of compound **5** alone and combination with 1 µM SAHA (Figure S1B, see Supplementary Materials). The compound **4** was the most active ligand among the quinoline candidates, with 30% inhibition of SH-SY5Y cell viability in the absence of SAHA and 20% additional cytotoxic effect in the presence of SAHA (Figure S1A, see Supplementary Materials). The designated compound displayed 25% reduction on MDA-MB-231 cells, while no SAHA enhancement effect was obtained against the same cell line (Figure S1B, see Supplementary Materials).

Overall, **1**, **3** and **4** were determined as the most promising compounds as SAHA enhancer and it was concluded that the indole heterocyclic systems **1** and **3** were found to be a potential target for the SAHA enhancement with the higher differential values between in the absence and presence of SAHA In order to further validate these compounds **1**, **3** and **4** as viable lead structures, it was essential to determine their toxicity towards normal and non-malignant cells. The most effective ligands were screened against the MRC-5 normal human lung fibroblast cells, as described for cytotoxic assay. This screen showed that the toxicity of **3** was found to be greater than **1** and **4** proposing that specificity of **3** against the normal and cancer cells was not different (Figure S2, see Supplementary Materials). The single treatment of compound **3** showed the cytotoxic activity with the values of 33% and 28% reduction against the SH-SY5Y and MDA-MB-231 cells respectively, while the toxicity of this compound against the MRC-5 cells was 26% and 30% greater than the SH-SY5Y and MDA-MB-231 cancer cells. However, compounds **1** and **4** displayed non-toxic behavior on the MRC-5 healthy human cells with values of 103% and 98% viable cells.

Based on the results of screening against the cancer cells and toxicity study on normal cells, the indole heterocyclic system **1** was identified as promising leads for further development on the enhancement of SAHA activity and compound **1** was also determined to be non-toxic across the healthy human cells. The main work described in this manuscript focused on the synthesis, characterization and in vitro biological evaluation of a series of targeted compounds based on tricyclic and tetracyclic dihydropyrano derivatives. The effectiveness of the novel compounds as single agents and in combination with a clinical dose of SAHA was determined against neuroblastoma and breast cancer cells. 

## 2. Results and Discussion

**Figure 2:**
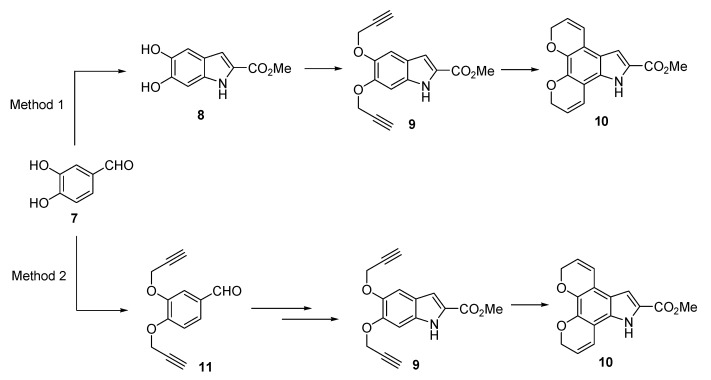
Two methods for the preparation of pyranoindole systems.

The preparation of dihydropyranoindole systems was achieved by two synthetic methods, and the generality of these pathways were discussed in this paper. 3,4-Dihydroxybenzaldehyde **7** has been given as an example for the representation of two possible methods which could be used for the preparation of the desired pyranoindole methyl 5,10-dihydro-7*H*-dipyrano [3,2-*e*:2′,3′-*g*]indole-6-carboxylate **10** (Figure 2). The first method was to prepare the compound **10** via methyl hydroxyindole-2-carboxylate **8** which could be prepared by the Hemetsberger indole synthesis [19]. Methyl hydroxyindole-2-carboxylate **8** on reacting with haloalkynes would give the alkyne indole-ether **9** which upon Claisen cyclization would afford the desired dihydropyranoindole **10**. As an alternative pathway, aryl ether benzaldehyde **11** would be prepared via simple alkylation of phenol **7** with haloalkynes and the Hemetsberger indole synthesis would then be applied to build the indole moiety. It is anticipated that thermal cyclization would yield the desired compound **10** in one step.

### 2.1. Preparation of Tetracyclic Dihydropyranoindole 10 Via (Method 1)

**Scheme 1:**
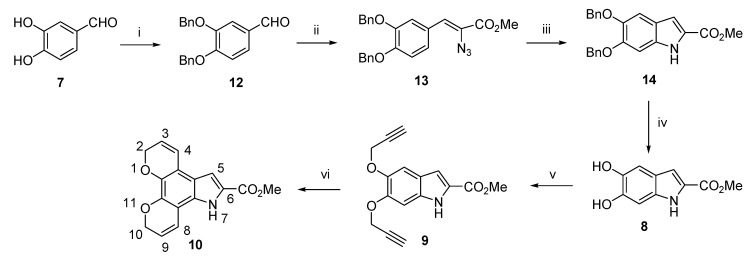
Reagents and conditions: (i) Benzyl bromide, K_2_CO_3_, DMF, reflux, overnight; (ii) Methyl azidoacetate, NaOMe, anhyd. MeOH, <−10 °C, 4 h; (iii) Xylene, reflux, 2 h, (iv) 5% Pd/C, H_2_, MeOH/THF, 2h, rt, (v) Propargyl bromide, K_2_CO_3_, acetone, reflux, 4 h, 61%, (vi) Chlorobenzene, reflux 2 h, 52%.

In the first synthetic method, methyl 5,6-dihydroxyindole-2-carboxylate **8** was generated via the Hemetsberger indole synthesis by benzyl-protected benzaldehydes and the subsequent deprotection afforded the corresponding hydroxyindoles. The 3,4-dihydroxybenzaldehyde **7** was reacted with benzyl bromide in the presence of potassium carbonate in acetone to afford the protected carbaldehydes **12** [20] in 88% yield (Scheme 1). Treatment of 3,4-dibenzyloxy benzaldehyde **12** with methyl azidoacetate in methanol, in the presence of strong basic environment (sodium methoxide) gave the vinyl azido intermediate **13** in 59% yield. Thermal decomposition of the arylazido **13** was performed by heating at reflux in xylene, generating the methyl 5,6-dibenzyloxyindole-2-carboxylate **14** in 71% yield. Hydrogenolysis of the benzyl group was carried out by treating the compound **14** with 5% *w*/*w* palladium on carbon under hydrogen atmosphere at room temperature for 2 h, to yield the desired methyl 5,6-dihydroxyindole-2-carboxylate **8** [21] in 77% yield. The dihydroxyindole **8** was reacted with propargyl bromide in the presence of potassium carbonate in acetone (Scheme 1). The desired dipropargyloxyindole intermediate **9** was prepared in 61% yield. The Claisen cyclization of **9** was explored in xylene, 1,2-dichlorobenzene and toluene. It was found that heating at reflux in chlorobenzene gave the optimum result in terms of the completion of the reaction as well as the yield and purity of the product. Using this approach, the desired tetracyclic dihydropyranoindole **10** was isolated in 51% yield.

**Figure 3:**
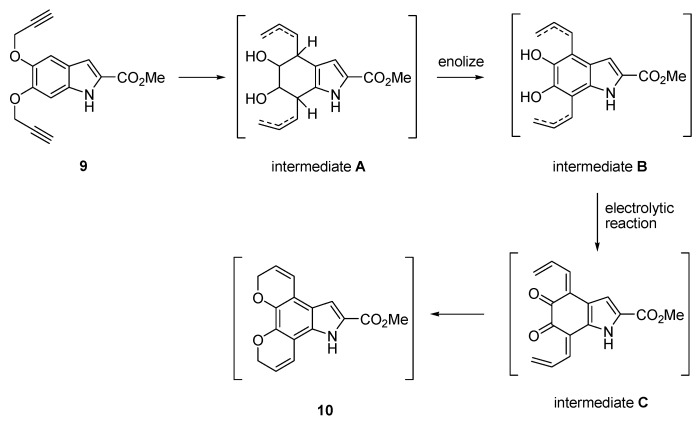
Possible reaction mechanism.

The possible reaction mechanism could be explained as in Figure 3. Methyl 5,6-bis(prop-2-yn-1-yloxy)-1*H*-indole-2-carboxylate **9** undergoes an initial Claisen rearrangement to generate intermediate **A**, which subsequently enolizes to produce 5,6-dihydroxyindole derivative (intermediate **B**). A double hydride shift in intermediate **B** gives the keto intermediate **C**, which undergoes an electrocyclic ring closing reaction to form methyl 5,10-dihydro-2*H*-dipyrano[3,2-*e*:2′,3′-*g*]indole-6-carboxylate **10**.

The ^1^H-NMR spectrum of compound **10** in CDCl_3_ showed a singlet at 3.96 ppm corresponding to the methyl ester protons, and two doublets of doublets at 4.91 and 4.96 ppm assigned to the two CH_2_ groups. A multiplet at 5.87–5.97 ppm corresponded to H3 and H9, while another multiplet at 6.72–6.80 ppm corresponded to H4 and H8. Furthermore, H3 appeared as a doublet at 7.19 ppm and the NH proton appeared as a broad singlet at δ 8.85 ppm. The DEPT 135 spectrum of compound **10** confirmed the structure of the molecule, displaying the loss of two CH carbon signals as a result of cyclization of the alkyne group as well as the appearance of two negative signals at 64.68 and 64.72 ppm corresponding to the methylene carbon atoms in the product.

### 2.2. Alternative Pathway for the Preparation of Tetracyclic Dihydropyranoindole 10 (Method 2)

**Scheme 2:**
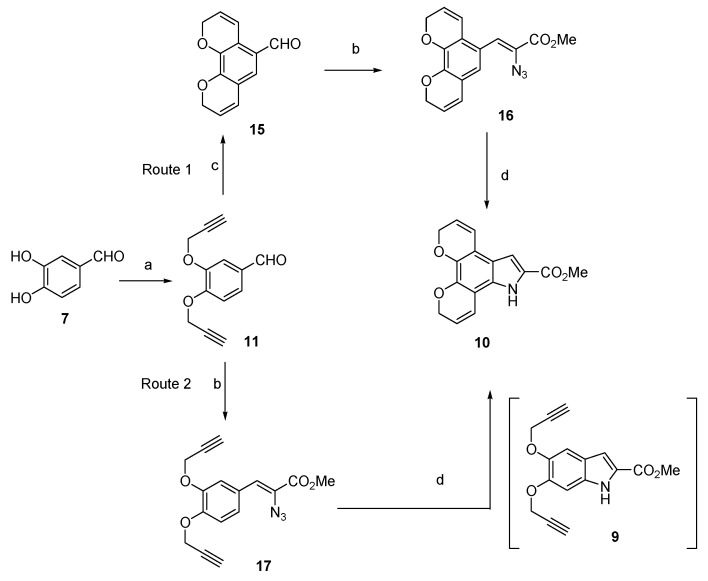
Reagents and conditions: (a) Propargyl bromide, K_2_CO_3_, acetone, reflux, 4 h, (b) Methyl azidoacetate, metallic Na, anhyd. MeOH, <−10 °C, 2 h (c) Chlorobenzene, reflux, 2 h, (d) Xylene, reflux, 4 h.

The alternative approach began with the reaction of 3,4-dihydroxybenzaldehyde **7** with propargyl bromide under basic conditions (potassium carbonate), which afforded the propargyloxy benzaldehyde **11** [22] in 87% yield. At this point, two synthetic strategies using benzaldehyde **11** as a key intermediate were envisioned (Scheme 2). The first route involved Claisen cyclization to build the dihydropyrano rings **15**, followed by the application of the Hemetsberger indole synthesis to construct the indole scaffold via the unsaturated azido intermediate **16** (Route 1). The cyclization of the aryl ether intermediate **11** was attempted by refluxing in high-boiling solvents such as xylene, toluene, 1,2-dichlorobenzene and chlorobenzene (Scheme 2). In all cases, the novel compound **15** was afforded in low yields, with unreacted starting material being recovered as the major product. The highest yield (35%) was obtained by the use of chlorobenzene. In order to synthesize the desired tetracyclic dihydropyranoindole **10**, the standard Hemetsberger indole synthesis was applied to new intermediate **15**. The condensation of benzaldehyde **15** with methyl azidoacetate was carried out at low temperature (ice-salt bath) in the presence of sodium methoxide. The addition of a small amount of crushed ice to the reaction mixture resulted in the isolation of azido ester **16** as an oily compound which was found to be unstable at room temperature, and was hence used in the next step without further purification. It was postulated that the presence of the dihydropyran ring caused the generation of an unstable azido ester intermediate. Thermal cyclization of the azido ester **16** was performed by heating at reflux in xylene, which afforded the desired dihydropyranoindole **10** in 62% yield.

In the second route, it was anticipated that the indole heterocyclic system could be constructed first, followed by the installation of dihydropyrano rings onto the indole moiety. Since the cyclization of both aryl ether and unsaturated azide ester moieties require refluxing conditions in a high-boiling solvent, it was predicted that the indole and dihydropyran rings could be prepared in one step from the key azide intermediate **17** (Route 2). The benzaldehyde **11** was condensed with methyl azidoacetate under strongly basic conditions at low temperature to generate the novel unsaturated stable azide intermediate **17** in 63% yield (Scheme 2). Since xylene was the common solvent for the generation of the indole moiety from the azido ester intermediate, the cyclization reaction was first carried out in refluxing xylene. As anticipated, the thermal cyclization of intermediate **17** resulted in both the decomposition of the azidocinnamate moiety and formation of the indole heterocyclic system, as well as the cyclization of the propargyloxy moiety to furnish the fused pyran ring in a single step. Hence, the desired tetracyclic dihydropyranoindole **10** was obtained in a yield of 72%.

Taken altogether, it was concluded that the first method is a less favorable synthetic pathway consisting of six steps to prepare the desired dihydropyranoindole and also generating the lowest of yield (51%) at the final step. In the case of the second method, route 1 was also found to be an unfavorable synthetic method for further synthesis due to the low-yielding cyclization step to generate the new tricyclic aldehyde **15**, the formation of the unstable azido intermediate **16** from the Hemestberger indole synthesis and the four step pathway for the desired dihydropyranoindole **10**. On the other hand, route 2 of the second strategy was deemed to be the most favorable synthetic route towards dihydropyranoindoles, as it contained three steps to afford the desired dihydropyranoindole and gave the highest yield of 72%. Thus, route 2 of the second method was chosen for further studies in this study.

### 2.3. The Preparation of Monomeric Hydropyrano System

**Scheme 3:**
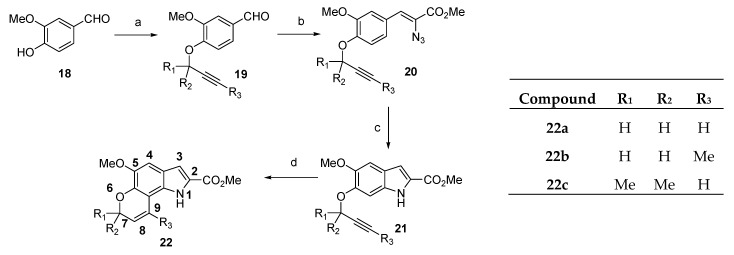
Reagents and conditions: (a) Propargyl bromide or 3-chloro-3-methylbut-1-yne or 1-bromobut-2-yne, K_2_CO_3_, acetone, reflux, 4 h, (b) Methyl azidoacetate, metallic Na, anhyd. MeOH, <−10 °C, 2 h (c) Xylene, reflux, 4 h, (d) Chlorobenzene, reflux, 2 h.

The synthetic strategy (Route 2) was extended to the synthesis of indole systems containing a single fused dihydropyran ring. Hence, 4-hydroxy-3-methoxy benzaldehyde **18** was reacted with propargyl bromide in the presence of potassium carbonate to give aryl ether **19a** [23] in 84% yield, which was then reacted with methyl azidoacetate under basic conditions to give azidocinnamate **20a** in 76% yield (Scheme 3). Interestingly, thermal cyclization of the **20a** in xylene afforded the corresponding propargyloxy indole **21a** in the yield of 68% instead of the expected dihydropyranoindole system **22a**. Further heating of indole **21a** in refluxing chlorobenzene afforded the desired dihydropyranoindole **22a** in 77% yield.

The ^1^H-NMR spectrum of compound **22a** in CDCl_3_ showed the presence of two singlets at 3.94 ppm and 3.96 ppm corresponding to the methoxy and methyl ester protons, and doublets of doublets at 4.96 ppm corresponding to the CH_2_ protons. Protons H8 and H9 appeared as multiplets in a range of 5.89–5.95 ppm and 6.73–6.79 ppm respectively. Furthermore, H4 appeared as a singlet at 7.02 ppm, while H3 was appeared as a doublet at 7.13 ppm (*J* = 2.1 Hz) due to its coupling with the NH proton, which resonated as a broad singlet at 8.94 ppm. The DEPT 135 spectrum showed the presence of a negative peak at 65.79 ppm corresponding to a methylene carbon atom. Furthermore, the spectrum revealed the absence of the alkyne CH signal due to cyclization of the alkyne moiety in indole **21a**.

The synthesis of tricyclic dihydropyranoindole systems containing methyl substituents on the fused dihydropyran ring at different positions was also investigated. To achieve this, 4-hydroxy-3-methoxybenzaldehyde **18** was reacted with 1-bromobut-2-yne and 3-chloro-3-methylbut-1-yne in the presence of potassium carbonate to give the aryl ether aldehydes **19b** [24] and **19c** [25] in 92% and 84% yields, respectively (Scheme 3). The aldehydes **19b** and **19c** were treated with methyl azidoacetate in the presence of sodium methoxide to give azidocinnamates **20b** and **20c** in yields of 61% and 62%. Finally, the thermal cyclization of the unsaturated azide **20b** and **20c** in refluxing xylene generated the desired product **22c** in 74% yields, while the indole ether **21b** was isolated in 67% yield. The desired dihydropyranoindole **22b** was obtained in 49% yield, by the further cyclization of **21b** in refluxing chlorobenzene.

In the ^1^H-NMR spectrum of methyl 5-methoxy-7,7-dimethyl-1,7-dihydropyrano[2,3-*g*]indole-2-carboxylate **22c**,the two methyl groups appeared as a singlet at 1.55 ppm, while H8 and H9 appeared as doublet signals at 5.71 and 6.66 ppm (*J* = 9.7 Hz). Furthermore, H4 appeared as a singlet at 7.01 ppm and H3 appeared as a doublet at 7.13 ppm (*J* = 2.1 Hz), confirming that cyclization occurred at C7. In the ^1^H-NMR spectrum of the cyclization product **22b**, the olefinic proton on the dihydropyran ring appeared as multiplet signals in the ranges of 5.65–5.68 ppm, due to coupling with the neighboring CH_2_ and CH_3_ groups. For compound **22b**, the O-CH_2_ protons resonated as a multiplet at 4.77 ppm and the methyl protons of the dihydropyran ring appeared as a quartet at 1.71 ppm.

**Scheme 4:**
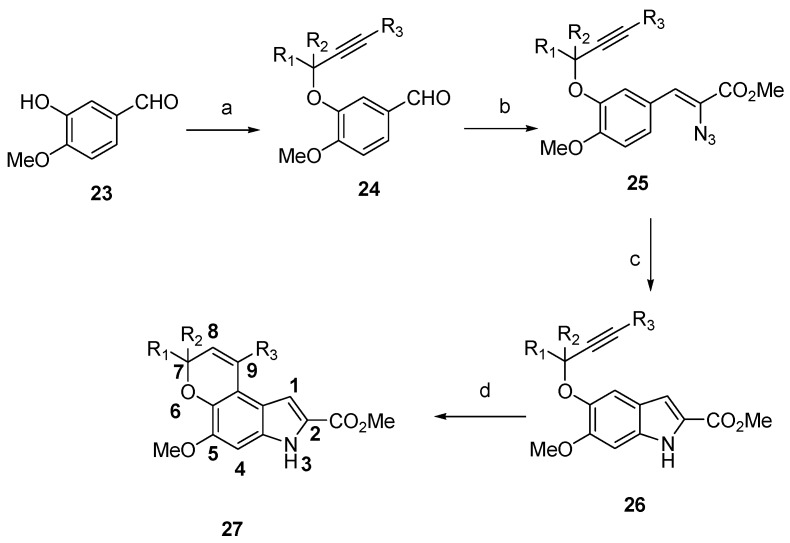
Reagents and conditions: (a) Propargyl bromide or 3-chloro-3-methylbut-1-yne or1-bromobut-2-yne or 3-phenylprop-2-yn-1-ol, K_2_CO_3_, acetone, reflux, 4 h, (b) Methyl azidoacetate, metallic Na, anhyd. MeOH, <−10 °C, 2 h (c) Xylene, reflux, 4 h, (d) Chlorobenzene, reflux, 2 h.

The same synthetic route was applied to 3-hydroxy-4-methoxy benzaldehyde **23** in order to generate an analogue with the dihydropyran ring fused at a different position. Thus, hydroxybenzaldehyde **23** was reacted with propargyl bromide in the presence of potassium carbonate to generate the intermediate ether **24a** [26] in 87% yield, which upon reaction with methyl azidoacetate in strongly basic conditions gave the azidocinnamate **25a** in 72% yield (Scheme 4). Heating the azidocinnamate **25a** in refluxing xylene gave the propargyloxy indole **26a** in 73% yield, which was then cyclized in chlorobenzene to afford the desired dihydropyranoindole **27a** in 74% yield.

The ^1^H-NMR spectrum of compound **27a** in CDCl_3_ showed two singlet signals at 3.92 and 3.93 ppm corresponding to the methoxy and methyl ester protons, and doublets of doublets at 4.90 ppm corresponding to the CH_2_ protons. H8 and H9 appeared as multiplets at 5.89–5.95 and 6.76–6.79 ppm respectively. The H4 appeared as a singlet at 6.77 ppm, while H1 appeared as a doublet at 7.17 ppm (*J* = 2.1 Hz). Moreover, the NH proton resonated as a broad singlet at δ 8.96 ppm. The formation of the desired dihydropyranoindole was further confirmed by DEPT 135 spectroscopy which revealed the presence of a negative peak at 65.71 ppm corresponding to the CH_2_ group accompanied by the disappearance of the signal corresponding to the CH group of the starting alkyne **26a**.

3-Hydroxy-4-methoxybenzaldehyde **23** was further treated with 1-bromobut-2-yne and 3-chloro-3-methylbut-1-yne under basic conditions to generate the corresponding intermediates **24b** [24] and **24c** [27] 89% and 82% yields, respectively (Scheme 4). The aryl ethers **24b** and **24c** were condensed with methyl azidoacetate in the presence of sodium methoxide to generate the azidoacrylates **25b** and **25c** in 64% and 62% yields respectively. Thermal decomposition of the unsaturated azides **25b** gave the indole ethers **26b** in 71%, while the desired hydropyrano compound **27c** was directly isolated in 68% yield. The desired dihydropyranoindoles **27b** was obtained in 54% yields respectively, by the cyclization of **26b** and in refluxing chlorobenzene. It was also of interest to construct new dihydropyranoindole systems containing substituents other than a methyl group on a pyran ring. The haloalkyne, 3-phenylprop-2-yn-1-ol, was treated with 3-hydroxy-4-methoxybenzaldehyde **23** in the presence of potassium carbonate to produce the alkylated carbaldehyde **24d** in 92% yield (Scheme 4). The reaction between the aryl ether **24d** with methyl azidoacetate afforded the unsaturated azido ester **25d** in 64% yield. Finally, heating compound **25d** in refluxing xylene afforded the corresponding indole ether **26d** in 74% yield, which further underwent thermal cyclization in chlorobenzene to furnish the desired dihydropyranoindole **27d** in 81% yield.

In the ^1^H-NMR spectrum of methyl 5-methoxy-7,7-dimethyl-3,7-dihydropyrano[3,2-*e*]indole-2-carboxylate **27c**, the two methyl groups appeared as a sharp singlet at 1.53 ppm. The H8 and H9 of the dihydropyran ring appeared as two doublets at 5.73 and 6.68 ppm that coupled to each other with a coupling constant of 9.7 Hz. H1 appeared as a doublet (*J* = 2.1 Hz) at 7.20 ppm while H4 was assigned as a singlet at 6.79 ppm. In the ^1^H-NMR spectrum of the cyclization product **27b** the olefinic protons on the dihydropyran ring appeared as multiplet signals in range of 5.69–5.72, due to coupling with the neighboring CH_2_ and CH_3_ groups. The characteristic O-CH_2_ protons resonated as a multiplet at 4.77 ppm, while the methyl group of the dihydropyran ring appeared at as a quartet 1.63 ppm. The ^1^H-NMR spectrum of compound **27d** displayed the O-CH_2_ protons as a doublet at 4.85 ppm with a coupling constant of 4.4 Hz, and the olefinic proton as a multiplet at 5.94–5.97 ppm. The aromatic protons of phenyl ring appeared as multiplet in the range of 7.34–7.44 ppm. The H4 and H1 appeared as two singlets at 5.98 and 6.88 ppm.

**Scheme 5:**
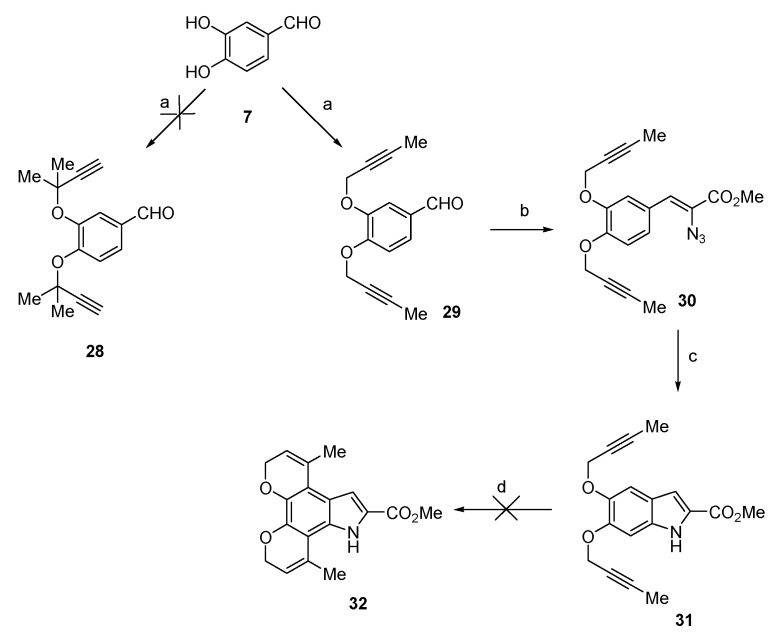
Reagents and conditions: (a) 1-Bromobut-2-yne or 3-Chloro-3-methyl-1-butyne, K_2_CO_3_, acetone, reflux, 4 h, (b) methyl azidoacetate, metallic Na, anhdyrous MeOH, −10 °C, 2 h (c) Xylene, reflux, 2 h, (d) Xylene, chlorobenzene, toluene, 1,2-dichlorobenzene reflux, overnight.

Similar attempts were made to the prepare methyl substituted tetracyclic dihydropyranoindole compounds from the 3,4-dihydroxybenzaldehyde **7**, 1-bromobut-2-yne and 3-chloro-3-methylbut-1-yne. The benzaldehyde **7** was alkylated with 2 equivalents of 1-bromobut-2-yne in the presence of potassium carbonate to generate the novel aryl ether **29** in 87% yield (Scheme 5). The benzaldehyde **29** was then treated with methyl azidoacetate under strongly basic conditions to generate the azido compound **30** in 63% yield, which subsequently underwent thermal decomposition in refluxing xylene to afford the indole ether **31** in 65% yield. However, the cyclization of **31** could not be achieved in a number of solvents, including chlorobenzene, toluene, xylene and 1,2-dichlorobenzene which resulted in the formation of decomposed reaction mixture. The reaction between 3,4-dihydroxybenzaldehyde **7** and 3-chloro-3-methylbut-1-yne was investigated in an attempt to produce the dipropargyloxybenzaldehyde intermediate **28** (Scheme 5). However, this reaction resulted in a black reaction mixture, presumably due to decomposition.

### 2.4. Biological Study

#### 2.4.1. Preliminary Biological Screening of Dihydropyranoindoles for SAHA Enhancement Activity

**Figure 4:**
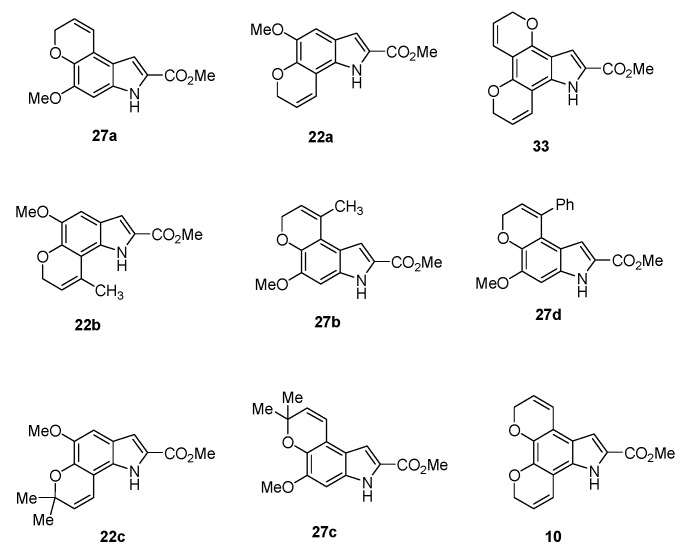
Synthesized and selected compounds.

Novel dihydropyranoindole analogues were screened to determine the levels of SAHA enhancement activity as well as their own cytotoxic profile against the SH-SY5Y and Kelly neuroblastoma cells and the MDA-MB-231 and MCF-7 breast adenocarcinoma cell lines using the Alamar blue (Resazurin) assay [18]. Briefly, the cells were allowed to attach for 24 h in a 96-well culture plate before being exposed to the ligands at a concentration of 10 µM in DMSO for 72 h, either in the presence or absence of SAHA. Comparative values for cell viability in each well were determined by a Wallac 1420 Victor III spectrophotometer, which measured light absorbance in each well at 570 nm. The mean and standard deviation (SD) values for each compound were calculated from at least three replicate experiments. The anticancer activity of the compounds was evaluated by comparison to a negative (DMSO) control. Figure 4 shows the tested novel dihydropyranoindoles synthesized in this study and the compound **33** was selected from the NK library. In this assay, the cell lines were exposed to SAHA, 10 μM of the compounds as well as the combination of SAHA with the compounds for 72 h.

According to the results from the screening, the neuroblastoma cells were found to be the more sensitive cells towards the treatment of new pyranoindoles, while these compounds showed a moderate effect on the breast cancer cell lines (Figures S3A,B and S4A,B, see Supplementary Materials). MDA-MB-231 cells were determined as the most resistant cell line for the single as well as the combination treatments (Figure S3A, see Supplementary Materials). The dihydropyranoindole **27c** exhibited the greatest cell viability inhibition with the values of 34% and 35% in the absence and presence of SAHA against the MDA-MB-231 cells. The SAHA enhancement was achieved by the additional reduction value of 20% across MDA-MB-231 viability. MCF-7 cells showed an identical pattern of sensitivity. However, most of the dihydropyranoindoles were able to reduce the viability by the value of 20% (Figure S3, see Supplementary Materials).

Compound **33** displayed the highest reduction with the 35% inhibition, while compounds **27c** and **22c** generated similar reductions with the values of 20% and 22% on the same cell line. Unfortunately the combination of pyranoindoles with 1 μM SAHA had very low SAHA enhancement by the average values of 5% across the MCF-7 cell lines.

It was observed that dihydropyranoindoles had a variety of impact on the viability of neuroblastoma cells (Kelly and SH-SY5Y) with the single and combination treatments (Figure S4A,B, see Supplementary Materials). Out of all the novel ligands synthesized, ligand **33** had the strongest cytotoxic activity with the reduction value of 50% at 10 μM, while ligands **27c** and **10** showed similar inhibition behaviors by the average reduction value of 32% against Kelly cells for the single treatment. The dihydropyranoindoles **27c** and **33** displayed the enhancement of SAHA cytotoxicity with the values of 24 and 22% respectively (Figure S4A, see Supplementary Materials). The value of 40% SAHA enhancement was obtained by the combination of compound **33**. However, the ligand **33** was considered as self-toxic compound due to the reduction value for the single treatment. The levels of individual cytotoxic efficiency against the SH-SY5Y were found to be higher than those observed on the Kelly cells (Figure S4B, see Supplementary Materials). The compounds **27d** and **10** showed the greatest cytotoxic activity reducing 44% and 56% of the cells respectively. Most importantly, the enhancement of SAHA activity was achieved by the treatment of almost all of the novel dihydropyranoindole analogues showing at least 20% additional reduction to SAHA cytotoxicity. Although the highest SAHA enhancement was obtained in the case of compound **10** with the value of 55%, it was not considered as a promising enhancer since the single treatment also generated the greatest reduction (56%) on the SH-SY5Y cell. The best enhancers were assigned as compounds **22a**, **27a**, **27c** and **33** with the reduction values of 25%, 29%, 30% and 31%respectively due to the lower toxicity but the higher enhancement effects on SAHA cytotoxicity.

#### 2.4.2. Determination of SAHA Enhancement Activity of Selected Dihydropyranoindoles at Lower Concentrations

Since the levels of SAHA enhancement were found to be promising, further investigations were carried out using five different concentrations (0.01, 0.1, 1, 10, 20 μM), using the Alamar blue (Resazurin) assay [18] to determine whether the lower concentrations would provide higher enhancement of the SAHA cytotoxicity or the cytotoxic manner would be dependent on the dose usage. The initial screening revealed that the single treatment of dihydropyranoindoles **33**, **27c** and **10** at a concentration of 10 μM showed the highest cytotoxic efficiency against the Kelly cells. The designated compounds were treated with the Kelly cells by the combinations of 0.5 μM SAHA with the five different concentrations (0.01, 0.1, 1, 10, 20 μM) and the results are shown at Figure S5A,C (see Supplementary Materials). In general, the single treatment of these compounds provided lower cytotoxic activity at the concentrations of 0.01, 0.1 and 1 μM compared with the values at 10 μM and the cell viability differentials between the absence and presence of SAHA were greater than those at 10 μM concentrations, suggesting that the designated compounds behave as suitable SAHA enhancers (Figure S5A,C, see Supplementary Materials). Similar SAHA enhancement pattern was observed at the concentrations of 0.01, 0.1 and 1 μM for all three compounds, while the combination of 1 μM compound **27c** or **10** with the 0.5 μM SAHA revealed the best SAHA enhancement with the values of 24% and 25% additional inhibition (Figure S5B,C, see Supplementary Materials).

In addition to those two compounds, compound **33** showed the greatest viability differentials with the values of 61%, 63% and 65% at the concentrations of 0.01, 0.1 and 1 μM and the desired SAHA enhancement was observed for all the concentrations. The lowest concentration of compound **33** (0.01 μM) revealed a remarkable reduction with the value of 25%, while the best combination for the highest SAHA enhancement was determined as a 10:0.5 ratio of compound and SAHA with the additional reduction value of 55% (Figure S5 A, see Supplementary Materials). It was concluded that the highest cell viability differentials was achieved by the use of nanomolar concentrations in the case of all three compounds and the best SAHA enhancer was determined as compound **33**.

Similarly, in the case of the SH-SY5Y cell line, further investigations were undertaken with **27a**, **33** and **27c** which were chosen due to their highest additional effects on the SAHA cytotoxicity with the lower toxicity behavior in the absence of SAHA. Compound **10** was also explored to determine its cytotoxic behavior as well as the SAHA enhancement potential at lower concentrations. The combinations of 1 μM SAHA with the five different concentrations (0.01, 0.1, 1, 10, 20 μM) of designated compounds were treated with the SH-SY5Y cells and the results are shown at Figure S6A–D (see Supplementary Materials).

The SAHA enhancement pattern was found to be identical on SH-SY5Y value for all cases of combinations of **27a** with SAHA. The highest viability differential was obtained in the ratio 1:10 of SAHA to compound with the reduction value of 35% (Figure S6A, see Supplementary Materials). The dose-dependent pattern of single treatments of **33** and **27c** suggested that these compounds displayed higher toxicity at 20 μM with the values of 45% and 55% reductions. Furthermore, the combinations of these compounds at the concentrations of 0.01, 0.1 and 1 μM with SAHA enhanced the SAHA cytotoxicity in a similar manner and the best combinations were found to be as the ratio 1:10 of SAHA to compound with the additional reduction values of 28% and 33% (Figure S6B,C, see Supplementary Materials). The greatest cytotoxicity was obtained at a concentration of 20 μM of **10** alone with the reduction values of 88% on SH-SY5Y cell viability and the ratio of 1:20 SAHA to compound combination provided 94% cell death (Figure S6D, see Supplementary Materials).

More importantly, at the lower concentrations (0.01, 0.1 and 1 μM) of **10**, the cytotoxicity was found to be lower with the average reduction value of 25%. Encouragingly, the biggest cell viability differential was obtained at the ratio of 1:10 SAHA to compound with the value of 44%, and the greatest SAHA enhancement was also found as 37% at the same combination. It was concluded that **10** was the most cytotoxic compound at higher concentration (20 μM, 88% reduction) and also it was found to be the best SAHA enhancer at the ratio of 1:10 SAHA to compound combination.

#### 2.4.3. SAR Study of Selected Dihydropyranoindoles

These observations suggested an important structure-activity relationship among **27a**, **33**, **27c** and **10**. That is, the incorporation of tetracyclic dihydropyranoindoles **33** and **10** resulted in lower cell viability reductions at higher concentrations (20 μM) and also this system was found to offer the best enhancement with the ratio of 1:10 SAHA to compound combinations against both neuroblastoma cancer cells (Kelly and SH-SY5Y). The SAR analysis revealed that the location of dihydropyran ring on the benzene ring was important for the cytotoxic efficiency of tricyclic dihydropyranoindoles, The compounds **27c**, an example of dihydropyrano[3,2-*e*]indole system, was found to be more potent than the dihydropyranoindole **22c** which is a member of dihydropyrano[2,3-*g*]indole scaffold against both neuroblastoma cells (Kelly and SH-SY5Y). In comparison of two tricyclic dihydropyranoindoles **27c** and **22c** at 20 μM, it was found that the compound **27c** reduced 20% more SH-SY5Y cell with the combination of 1 μM SAHA than compound **22c**, while the 20% SAHA enhancement was achieved using the combination of compound **27c** and SAHA but no SAHA enhancement was found with the compound **22c** in the case of Kelly cells. Furthermore, the dihydropyrano[2,3-*g*]indole analogues **22a** and **22b** displayed the lowest reduction on Kelly cells in the presence and absence of SAHA. Similar results were observed in the case of SH-SY5Y cells with the exception of 21% additional reduction on SAHA enhancement obtained by the use of **22a**.

#### 2.4.4. Toxicity Study Against Normal Cells

While selected dihydropyranoindoles showed potent SAHA enhancement activity against Kelly and SH-SY5Y neuroblastoma and MCF-7 and MDA-MB-231 breast cancer cells, they must be markedly less toxic against the healthy cells in order to be considered as potential SAHA enhancers. Thus, the dihydropyranoindoles **27a**, **33**, **27c** and **10** were also examined for their toxicity effects on the MRC-5 and WI-38 lung fibroblasts in order to determine whether these compounds exhibited selectivity for tumor cells (Figure S7A–D, see Supplementary Materials). Comparison of the cytotoxic activity and toxicity levels of the dihydropyranoindoles against cancer cells and the healthy cells revealed that the cancer cells were more sensitive to the dihydropyranoindoles compared to the normal cells. The toxicity of 1 μM of SAHA reduced the viability of MRC-5 and WI-38 cells with the value of 9% and 24% respectively, while **27a** alone displayed a similar pattern of toxicity against normal cells with values of 10% for MRC-5 and 22% for WI-38 at a concentration of 10 μM (Figure S7A, see Supplementary Materials). The viability of MRC-5 and WI-38 were reduced 21% and 16%, 3% and 26% by the use of **33** and **10** alone (Figure S7B,C, see Supplementary Materials), while the highest toxicity levels were obtained in the case of **27c** with the reduction values of 20% and 43% (Figure S7D, see Supplementary Materials). The combination treatments were found to be quite identical to the reduction values of single treatments of each compound. These observations demonstrated that **27a**, **33** and **10** were either less toxic or slightly less toxic than SAHA, while **27c** displayed more toxicity than SAHA for both normal cells.

## 3. Materials and Methods

### 3.1. General Information

Commercially available reagents were purchased from Fluka (Sydney, NSW, Australia), Aldrich (Sydney, NSW, Australia), Acros Organics (Morris Plains, NJ, USA), Alfa Aesar (Lancashire, UK) and Lancaster (Lancashire, UK) and purified if necessary. The synthetic procedures have been reported for all compounds as general methods and appropriate references have been given for known compounds. ^1^H (300 MHz) and ^13^C-NMR (75 MHz) spectra were obtained in the designated solvents on a DPX 300 spectrometer (Bruker, Sydney, NSW, Australia). Melting points were measured using a Mel-Temp melting point apparatus and are uncorrected. Infrared spectra were recorded on Avatar Series FT-IR spectrophotometer as KBr disks (Thermo Nicolet, Waltham, MA, USA). Ultraviolet spectra were measured using a Cary 100 spectrophotometer (Varian, Santa Clara, CA, USA) in the designated solvents and data reported as wavelength (λ) in nm and adsorption coefficient (ε) in cm^−1^M^−1^. High-resolution [ESI] mass spectra were recorded by the UNSW Bioanalytical Mass Spectrometry Facility, on an Orbitrap LTQ XL (Thermo Scientific, Waltham, MA, USA) ion trap mass spectrometer using a nanospray (nano-electrospray) ionization source.

#### 3.1.1. GP-1: General Procedure for the Benzylation of Dihydroxybenzaldehydes

A solution of hydroxybenzaldehyde (1 equiv.), anhydrous potassium carbonate (per hydroxyl group, 1 equiv.) and benzyl bromide (per hydroxyl group, 1 equiv.) in anhydrous DMF (100 mL) was heated at reflux until TLC analysis showed consumption of the starting aldehyde (9 h). Upon cooling to room temperature, the reaction mixture was diluted with water (300 mL) and the resulting precipitate was collected via filtration and washed with water (2 × 250 mL). Upon drying, the residue was recrystallized from dichloromethane and *n*-hexane to give the title compound.

#### 3.1.2. GP-2: General Procedure for the Preparation of Vinyl-Azido Intermediates

A solution of sodium methoxide was prepared via the portion-wise addition of metallic sodium (17 equiv.) to anhydrous methanol (30 mL) with stirring under nitrogen. A dropping funnel was attached to the reaction flask and charged with the corresponding aldehyde (1 equiv.) and methyl azidoacetate (10 equiv.) in methanol (15 mL). The contents of the funnel were added dropwise to the sodium methoxide solution over 1.5 h under a nitrogen atmosphere. Once the addition was completed, the reaction mixture was warmed to 5 °C, where it remained for 4 h. The heterogeneous mixture was poured into crushed ice. The resulting precipitate was filtered, washed with water and dried to give the title compound. The crude product was used in the next step without further purification.

#### 3.1.3. GP-3: General Procedure for the Preparation of Methyl Benzyloxyindole-2-carboxylates

The vinyl azido intermediate (6.66 mmol) was dissolved in xylene (50 mL) and the reaction mixture was heated under reflux for 6 h. After refluxing, the solvent was evaporated under reduced pressure and the remaining residue was extracted with boiling hexane. Upon cooling, the resulting solid was filtered to give crude product which was recrystallized from dichloromethane and *n*-hexane to give the title compound.

#### 3.1.4. GP-4: General Procedure for the Hydrogenolysis of Methyl Dibenzyloxyindole-2-carboxylate

After vacuum/H_2_ cycles to remove air from the reaction flask, a stirred mixture of benzyloxyindole (1 mmol) and 5% Pd/C (10% *w*/*w*) in THF/MeOH (1:1, 15 mL) was exposed to a hydrogen atmosphere (1 atm) and stirred at room temperature for 2 h. The reaction mixture was filtered through a pad of Celite^®^ and the filtrate was then concentrated under reduced pressure. The crude product was purified by flash column chromatography (SiO_2_), eluted with dichloromethane to give the title compounds.

#### 3.1.5. GP-5: General Procedure for the Synthesis of Propargyloxybenzaldehydes:

Propargyl bromide (per hydroxyl group, 1.2 equiv.) was added to a mixture of potassium carbonate (per hydroxyl group, 1 equiv.) and hydroxybenzaldehyde (1 equiv.) in acetone. The reaction mixture was heated under reflux with stirring until no more starting material remained (~30 h). The reaction mixture was cooled to room temperature and Et_2_O (100 mL) was added. The ethereal solution was washed with NaOH (1 N, 3 × 50 mL). The organic layer was dried over MgSO_4_, concentrated under reduced pressure and recrystallized from dichloromethane/*n*-hexane to give the compound.

#### 3.1.6. GP-6 General Procedure for the Synthesis of Dihydropyranoindoles:

A solution of alkyne indole ethers (1.04 mmol) in chlorobenzene (20 mL) was heated under reflux until TLC analysis showed consumption of the starting indole (12 h). The heating was discontinued and the solvent was evaporated under reduced pressure. The crude product was purified using flash column chromatography (SiO_2_), eluted with 30% dichloromethane/*n*-hexane, to give the dihydropyranoindole.

*Methyl 2-azido-3-(3,4-dibenzyloxyphenyl)-propenoate* (**13**) The *title* compound was prepared as described in GP-2 from 3,4-dibenzyloxybenzaldehyde (**12**) (2.95 g, 9.3 mmol) and methyl azidoacetate (10.69 g, 93 mmol) in anhydrous methanol (30 mL) to give the product (2.27 g, 59%) as a pale yellow granular solid; m.p. 114–116 °C; IR (KBr): *v*_max_ 2917, 2119, 1701, 1683, 1590, 1508, 1432, 1379, 1233, 1201, 1130, 999, 802, 728 cm^−1^; UV-vis (CH_3_CN): λ_max_325 (24,500); ^1^H-NMR: (300 MHz, CDCl_3_): δ 3.91 (s, 3H, CO_2_Me), 5.25 (s, 4H, 2 × O-CH_2_), 6.83 (s, 1H, CH=C), 7.35–7.52 (m, 12H, ArH), 7.64 (d, *J* = 2.2 Hz, 1H, H2′); ^13^C-NMR: (75.6 MHz, CDCl_3_): δ 52.7 (CO_2_Me), 70.9 (CH_2_), 71.3 (CH_2_), 113.8 (C2′), 116.6 (C5′), 123.3 (CH=C), 125.4 (C6′), 125.7 (ArC), 126.6 (ArC), 127.1 (2 × ArC), 127.2 (2 × ArC), 127.8 (ArC), 127.9 (ArC), 128.5 (2 × ArC), 128.6 (2 × ArC), 136.8 (C1′), 137.1 (CH=C), 148.3 (C4′), 150.1 (C3′), 164.1 (CO_2_Me); HRMS (+ESI): Found *m*/*z* 438.1439 [M + Na]^+^, C_24_H_21_N_3_O_4_Na requires 438.1439.

*Methyl 5,6-dibenzyloxyindole-2-carboxylate* (**14**) The *title* compound was prepared as described in GP-3 from methyl 2-azido-3-(3′,4′-dibenzyloxyphenyl)propenoate (**13**) (2.76 g, 6.66 mmol) in xylene (50 mL) to give the product (1.84 g, 71%) as a yellow granular solid; m.p. 148–150 °C; IR (KBr): *v*_max_ 3309, 2942, 2871, 2113, 1680, 1627, 1519, 1488, 1452, 1353, 1288, 1246, 1208, 1144, 1000, 918, 905, 839, 794 cm^−1^; UV-vis (CH_3_CN): λ_max_ 208 nm (ε 72,400 cm^−1^ M^−1^), 320 (31,700); ^1^H-NMR (300 MHz, CDCl_3_): δ 3.93 (s, 3H, CO_2_Me), 5.21 (s, 2H, O-CH_2_), 5.24 (s, 2H, O-CH_2_), 7.00 (s, 1H, H4), 7.01 (s, 1H, H7), 7.23 (d, *J* = 2.1 Hz, 1H, H3), 7.33–7.51 (m, 10H, ArH), 8.73 (bs, 1H, NH); ^13^C-NMR (75.6 MHz, CDCl_3_): 51.8 (CO_2_Me), 71.3 (CH_2_), 72.0 (CH_2_), 97.0 (C7), 107.0 (C4), 108.8 (C3), 121.0 (ArC), 126.0 (C2), 127.0 (ArC), 127.2 (2 × ArC), 127.3 (ArC), 127.7 (2 × ArC), 127.8 (ArC), 128.4 (ArC), 128.5 (ArC), 132.4 (ArC), 137.0 (ArC), 137.4 (2 × ArC), 145.8 (C5), 149.9 (C6), 162.1 (CO_2_Me); HRMS (+ESI): Found *m*/*z* 410.1364 [M + Na]^+^, C_24_H_21_NO_4_Na requires 410.1363.

*Methyl 5,6-dihydroxyindole-2-carboxylate* (**8**) The *title* compound was prepared as described in GP-4 from methyl 5,6-dibenzyloxyindole-2-carboxylate (**14**) (0.387 g, 1.0 mmol) and 5% Pd/C catalyst (40 mg) in methanol/THF mixture (15 mL) to give the product (159 mg, 77%) as yellow solid; m.p. 256–258 °C; IR (KBr): *v*_max_ 3437, 3315, 2953, 2107, 1653, 1632, 1531, 1506, 1437, 1311, 1283, 1230, 1198, 1139, 992, 937, 849, 825, 767 cm^−1^; UV-vis (CH_3_CN): λ_max_ 203 nm (ε 42,100 cm^−1^ M^−1^), 318 (27,000);^1^H-NMR (300MHz, *d*_6_-DMSO): δ 3.82 (s, 3H, CO_2_Me), 6.79 (d, *J* = 0.8 Hz, 1H, H4), 6.88 (s, 1H, H3), 6.90 (d, *J* = 0.8 Hz, 1H, H7), 8.84 and 9.17 (bs, each 1H, OH), 11.28 (bs, 1H, NH); ^13^C-NMR (75.6 MHz, CDCl_3_): 52.0 (CO_2_Me), 97.3 (C7), 105.4 (C4), 108.4 (C3), 120.3 (ArC), 125.0 (C2), 133.2 (ArC), 142.3 (C5), 146.7 (C6), 162.5 (CO_2_Me); HRMS (+ESI): Found *m*/*z* 230.0423 [M + Na]^+^, C_10_H_9_NO_4_Na requires 230.0424.

*Methyl 5,6-bis(prop-2-yn-1-yloxy)-1H-indole-2-carboxylate* (**9**) The *title* compound was prepared as described in GP-5 from methyl 5,6-dihydroxyindole-2-carboxylate (**8**) (993 mg, 4.8 mmol), potassium carbonate (1.32 g, 9.6 mmol) and propargyl bromide (1.36 g, 10.6 mmol) in acetone to give the product (828 mg, 61%) as a yellow solid;m.p. 170–172 °C; IR (KBr): *ν*_max_ 3332, 3285, 2939, 2925, 2865, 2292, 2108, 1720, 1684, 1625, 1520, 1480, 1435, 1380, 1365, 1280, 1247, 1203, 1150 1024, 986, 892, 819, 805, 759, 720, 672 cm^−1^; UV-vis (CH_3_CN): λ_max_ 209 nm (ε 28,300 cm^−1^ M^−1^), 313 (18,000); ^1^H-NMR (300 MHz, CDCl_3_): δ 2.55 (t, *J* = 1.6 Hz, 1H, CH≡C), 2.57 (t, *J* = 1.6 Hz, 1H, CH≡C), 3.95 (s, 3H, CO_2_Me), 4.82 (d, *J* = 2.4 Hz, 2H, O-CH_2_), 4.85 (d, *J* = 2.4 Hz, 2H, O-CH_2_), 7.10 (d, *J* = 0.7 Hz, 1H, H7), 7.16 (q, *J* = 0.9 Hz, 1H, H3), 7.31 (s, 1H, H4), 8.86 (bs, 1H, NH), ^13^C-NMR (75.6 MHz, CDCl_3_): δ51.8 (CO_2_Me), 57.0 (O-CH_2_), 57.5 (O-CH_2_), 75.7 (C≡CH), 75.7 (C≡CH), 78.6 (C≡CH), 79.1 (C≡CH), 97.2 (C7), 107.2 (C4), 108.9 (C3), 121.3 (aryl C), 126.4 (C2), 132.4 (aryl C), 144.2 (C4), 148.2 (C5), 162.1 (CO_2_Me); HRMS (+ESI): Found *m*/*z* 306.0736 [M + Na]^+^, C_16_H_13_NO_4_Na requires 306.0737.

*2,9-Dihydropyrano[3,2-h]chromene-5-carbaldehyde* (**15**) The *title* compound was prepared as described in GP-6 from 3,4-bis(prop-2-yn-1-yloxy)benzaldehyde (**11**) (214 mg, 1.04 mmol) in chlorobenzene (20 mL) to give the product (77 mg, 35%) as a white solid; m.p. 102–104 °C; IR (KBr): *ν*_max_ 1680, 1570, 1490, 1440, 1340, 1301, 1205, 1102, 995, 945, 900, 898, 861, 745 cm^−1^; UV-vis (CH_3_CN): λ_max_ 225 nm (ε 27,400 cm^−1^ M^−1^), 268 (18,800), 305 (17,500); ^1^H-NMR (300 MHz, CDCl_3_): δ 4.40 (dd, *J* = 3.0, 1.4 Hz, 2H, O-CH_2_), 4.79 (dd, *J* = 3.0, 1.4 Hz, 2H, O-CH_2_), 6.97 (d, *J* = 8.7 Hz, 2H, H4 and H7), 7.37 (s, 1H, H6), 7.40–7.42 (m, 2H, H3 and H8), 9.80 (s, 1H, CHO); ^13^C-NMR (75.6 MHz, CDCl_3_): δ71.3 (O-CH_2_), 71.5 (O-CH_2_), 119.6 (C6), 120.6 (aryl C), 121.6 (C4), 121.8 (C7), 125.3 (aryl C), 126.5 (C3), 126.9 (C8), 127.1 (C5), 147.9 (aryl C), 154.3 (aryl C), 190.7 (CHO); HRMS (+ESI): Found *m/z* 237.0533 [M + Na]^+^, C_13_H_10_O_3_Na requires 237.0528.

*3,4-Bis(but-2-yn-1-yloxy)benzaldehyde* (**29**) The *title* compound was prepared as described in GP-5 from 3,4-dihydroxybenzaldehyde (7) (660 mg, 4.8 mmol), potassium carbonate (1.32 g, 9.6 mmol) and 1-bromobut-2-yne (1.40 g, 10.6 mmol) in acetone to give the product (1.01 g, 87%) as a white solid; m.p. 86–88 °C; IR (KBr): *ν*_max_ 1688, 1582, 1499, 1431, 1375, 1248, 1201, 1125, 987, 920, 857, 797, 729 cm^−1^; UV-vis (CH_3_CN): λ_max_ 228 nm (ε 31,500 cm^−1^ M^−1^), 272 (22,200), 303 (14,800); ^1^H-NMR (300 MHz, CDCl_3_): δ 1.85–1.87 (m, 6H, 2 × C-CH_3_), 4.79 (q, *J* = 2.3 Hz, 2H, O-CH2), 4.82 (q, *J* = 2.3 Hz, 2H, O-CH2), 7.17 (d, *J* = 8.2 Hz, 1H, H5), 7.52 (dd, *J* = 8.2, 1.9 Hz, 1H, H6), 7.57 (d, *J* = 1.9 Hz, 1H, H2), 9.89 (s, 1H, CHO); ^13^C-NMR (75.6 MHz, CDCl_3_): δ 3.6 (C-CH_3_), 3.7 (C-CH_3_), 57.2 (2 × O-CH_2_), 73.1 (C-CH_3_), 73.3 (C-CH_3_), 84.6 (C≡C(CH_3_)), 84.9 (C≡C(CH_3_)), 112.0 (C5), 112.7 (C2), 126.4 (C6), 130.3 (aryl C), 147.9 (C4′), 152.9 (C3′), 190.8 (CHO); HRMS (+ESI): Found *m*/*z* 265.0840 [M + Na]^+^, C_15_H_14_O_3_Na requires 265.0835.

*4-Methoxy-3-((3-phenylprop-2-yn-1-yl)oxy)benzaldehyde* (**24d**) The *title* compound was prepared as described in GP-5 from 3-hydroxy-4-methoxybenzaldehyde **23** (730 mg, 4.8 mmol), potassium carbonate (662 mg, 4.8 mmol) and 3-phenylprop-2-yn-1-yl-4-methylbenzenesulfonate (1.66 g, 5.8 mmol) in acetone to give the product (1.17 g, 92%) as a white solid; m.p. 112–114 °C; IR (KBr): *ν*_max_ 1673, 1579, 1506, 1430, 1378, 1256, 1221, 1165, 1130, 1012, 931, 874, 810, 754, 686 cm^−1^; UV-vis (CH_3_CN): λ_max_ 232 nm (ε 45,500 cm^−1^ M^−1^), 272 (21,500), 302 (14,800); ^1^H-NMR (300 MHz, CDCl_3_): δ 4.00 (s, 3H, OMe), 5.08 (s, 2H, O-CH2), 7.03 (d, *J* = 8.2 Hz, 1H, H5), 7.30–7.33 (m, 3H, ArH), 7.34–7.45 (m, 2H, ArH), 7.54 (dd, *J* = 8.2, 1.9 Hz, 1H, H6), 7.68 (d, *J* = 1.9 Hz, 1H, H2), 9.90 (s, 1H, CHO); ^13^C-NMR (75.6 MHz, CDCl_3_): δ 56.2 (OMe), 57.5 (O-CH_2_), 83.0 (C≡C(Ph)), 88.0 (C-Ph), 110.9 (C5), 112.1 (C2), 122.1 (aryl C), 127.1 (2 × aryl CH), 127.8 (aryl CH), 128.8 (C6), 130.0 (aryl C), 131.9 (2 × aryl CH), 147.5 (C4), 155.0 (C3), 190.7 (CHO); HRMS (+ESI): Found *m*/*z* 289.0837 [M + Na]^+^, C_17_H_14_O_3_Na requires 289.0835.

*Methyl (Z)-2-azido-3-(3,4-bis(prop-2-yn-1-yloxy)phenyl) acrylate* (**17**) The *title* compound was prepared as described in GP-2 from 3,4-bis(prop-2-yn-1-yloxy)benzaldehyde (**11**) (558 mg, 2.6 mmol) and methyl azidoacetate (2.99 g, 26.0 mmol) in anhydrous methanol (30 mL) to give the product (582 mg, 63%) as a pale yellow granular solid; m.p. 114–116 °C; IR (KBr): *ν*_max_ 2954, 2123, 1709, 1594, 1506, 1433, 1378, 1239, 1133, 1081, 1009, 793, 745, 683 cm^−1^; UV-vis (CH_3_CN): λ_max_ 327 nm (ε 19,100 cm^−1^ M^−1^); ^1^H-NMR (300 MHz,CDCl_3_): δ 2.57 (t, *J* = 2.4 Hz, 1H, CH≡C), 2.59 (t, *J* = 2.4 Hz, 1H, CH≡C), 3.92 (s, 3H, CO_2_Me), 4.83 (d, *J* = 2.4 Hz, 4H, 2 × O-CH2), 6.91 (s, 1H, CH=C), 7.09 (d, *J* = 8.5 Hz, 1H, H5′), 7.44 (dd, *J* = 8.5, 2.0 Hz, 1H, H6′), 7.77 (d, *J* = 2.0 Hz, 1H, H2′), ^13^C-NMR (75.6 MHz, CDCl_3_): δ 52.8 (CO_2_Me), 56.6 (OMe), 57.0 (2 × O-CH_2_), 76.1 (C≡CH), 76.2 (C≡CH), 77.2 (C≡CH), 77.4 (C≡CH), 113.8 (C5′), 116.6 (C2′), 123.9 (CH=C), 125.3 (C6′), 125.6 (aryl C), 146.9 (aryl C), 148.5 (aryl C), 164.1 (CO_2_Me); HRMS could not be determined due to the unstable properties of the compound

*Methyl (Z)-2-azido-3-(4-methoxy-3-(prop-2-yn-1-yloxy)phenyl)acrylate* (**25a**) The *title* compound was prepared as described in GP-2 from 4-methoxy-3-(prop-2-yn-1-yloxy)benzaldehyde (24a) (494 mg, 2.6 mmol) and methyl azidoacetate (2.99 g, 26.0 mmol) in anhydrous methanol (30 mL) to give the product (537 mg, 72%) as a pale yellow granular solid; m.p. 116–118 °C; IR (KBr): *ν*_max_ 2951, 2101, 1698, 1592, 1507, 1432, 1379, 1254, 1214, 1139, 1084, 1016, 809, 745 cm^−1^; UV-vis (CH_3_CN): λ_max_ 235 nm (ε 16,600 cm^−1^ M^−1^), 330 (27,100); ^1^H-NMR (300 MHz, CDCl_3_): δ 2.58 (t, *J* = 2.4 Hz, 1H, CH≡C), 3.94 (s, 3H, OMe), 3.94 (s, 3H, CO_2_Me), 4.83 (d, *J* = 2.4 Hz, 2H, O-CH2), 6.90 (s, 1H, CH=C), 6.93 (d, *J* = 8.5 Hz, 1H, H5′), 7.43 (dd, *J* = 8.5, 2.0 Hz, 1H, H6′), 7.76 (d, *J* = 2.0 Hz, 1H, H2′); ^13^C-NMR (75.6 MHz, CDCl_3_): δ 52.8 (CO_2_Me), 55.9 (OMe), 56.8 (O-CH_2_), 76.0 (C≡CH), 77.4 (C≡CH), 111.2 (C5′), 116.1 (C2′), 123.4 (CH=C), 125.6 (C6′), 125.0 (aryl C), 126.1 (CH=C), 146.3 (C4′), 150.8 (C3′), 164.1 (CO_2_Me); HRMS (+ESI): Found *m*/*z* 310.0800 [M + Na]^+^, C_14_H_13_N_3_O_4_Na requires 310.0798.

*Methyl (Z)-2-azido-3-(3-methoxy-4-(prop-2-yn-1-yloxy) phenyl)acrylate* (**20a**) The *title* compound was prepared as described in GP-2 from 3-methoxy-4-(prop-2-yn-1-yloxy)benzaldehyde (**19a**) (494 mg, 2.6 mmol) and methyl azidoacetate (2.99 g, 26.0 mmol) in anhydrous methanol (30 mL) to give the product (567 mg, 76%) as a pale yellow granular solid; m.p. 120–122 °C; IR (KBr): *ν*_max_ 2920, 2122, 1704, 1592, 1509, 1450, 1345, 1243, 1206, 1141, 1008, 922, 804, 764 cm^−1^; UV-vis (CH_3_CN): λ_max_ 323 nm (ε 17,500 cm^−1^ M^−1^); ^1^H-NMR (300 MHz, CDCl_3_): δ 2.56 (t, *J* = 2.4 Hz, 1H, CH≡C), 3.94 (s, 3H, OMe), 3.95 (s, 3H, CO_2_Me), 4.83 (d, *J* = 2.4 Hz, 2H, O-CH2), 6.91 ( s, 1H, CH=C), 7.06 (d, *J* = 8.5 Hz, 1H, H5′), 7.43 (dd, *J* = 8.5, 1.9 Hz, 1H, H6′), 7.56 (d, *J* = 1.9 Hz, 1H, H2′); ^13^C-NMR (75.6 MHz, CDCl_3_): δ 52.8 (CO_2_Me), 55.9 (OMe), 56.5 (O-CH_2_), 76.2 (C≡CH), 77.4 (C≡CH), 113.3 (C5′), 113.5 (C2′), 123.7 (CH=C), 124.4 (C6′), 125.5 (aryl C), 127.3 (CH=C), 147.9 (C3′), 149.1 (C4′), 164.1 (CO_2_Me); HRMS could not be determined due to the unstable properties of the compound.

*Methyl (Z)-2-azido-3-(3,4-bis(but-2-yn-1-yl)oxy)phenyl) acrylate* (**30**) The *title* compound was prepared as described in GP-2 from 3,4-bis(but-2-yn-1-yloxy)benzaldehyde (**29**) (629 mg, 2.6 mmol) and methyl azidoacetate (2.99 g, 26.0 mmol) in anhydrous methanol (30 mL) to give the product (555 mg, 63%) as a pale yellow granular solid; m.p. 124–126 °C; IR (KBr): *ν*_max_ 2918, 2119, 1712, 1687, 1590, 1506, 1432, 1373, 1314, 1243, 1128, 992, 804, 745 cm^−1^; UV-vis (CH_3_CN): λ_max_ 307 nm (ε 14,500 cm^−1^ M^−1^); ^1^H-NMR (300 MHz, CDCl_3_): δ 1.87 (t, *J* = 2.3 Hz, 6H, 2 × C-CH_3_), 3.93 (s, 3H, CO_2_Me), 4.79 (q, *J* = 2.3 Hz, 2H, O-CH_2_), 4.82 (q, *J* = 2.3 Hz, 2H, O-CH_2_), 6.91 (s, 1H CH=C), 7.05 (d, *J* = 8.5 Hz, 1H, H5′), 7.44 (dd, *J* = 8.5, 1.8 Hz, 1H, H6′), 7.73 (d, *J* = 2.0 Hz, 1H, H2′); ^13^C-NMR (75.6 MHz, CDCl_3_): δ 3.7 (2×C-CH_3_), 52.8 (CO_2_Me), 57.1 (OMe), 57.2 (O-CH_2_), 57.5 (O-CH_2_), 73.9 (2×C-CH_3_), 84.2 (C≡C(CH_3_)), 84.9 (C≡C(CH_3_)), 113.2 (C5′), 116.0 (C2′), 125.3 (CH=C), 125.8 (C6′), 126.5 (aryl C), 126.6 (CH=C), 147.9 (C4′), 148.8 (C3), 164.2 (CO_2_Me); HRMS could not be determined due to the unstable properties of the compound.

*Methyl (Z)-2-azido-3-(3-(but-2-yn-1-yloxy)-4-methoxyphenyl) acrylate* (**25b**) The *title* compound was prepared as described in GP-2 from 3-(but-2-yn-1-yloxy)-4-methoxybenzaldehyde (**24b**) (530 mg, 2.6 mmol) and methyl azidoacetate (2.9 g, 26.0 mmol) in anhydrous methanol (30 mL) to give the product (500 mg, 64%) as a pale yellow granular solid; m.p. 96–98 °C; IR (KBr): *ν*_max_ 2951, 2101, 1698, 1592, 1507, 1431, 1379, 1254, 1139, 1016, 810, 745 cm^−1^; UV-vis (CH_3_CN): λ_max_ 327 nm (ε 25,700 cm^−1^ M^−1^); ^1^H-NMR (300 MHz, CDCl_3_): δ 1.89 (t, *J* = 2.3 Hz, 3H, CH_3_), 3.93 (s, 3H, OMe), 3.94 (s, 3H, CO_2_Me), 4.78 (q, *J* = 2.3 Hz, 2H, O-CH_2_), 6.91 (d, *J* = 8.5 Hz, 1H, H5′), 6.92 (s, 1H, CH=C), 7.41 (dd, *J* = 8.5, 2.0 Hz, 1H, H6′), 7.73 (d, *J* = 2.0 Hz, 1H, H2′); ^13^C-NMR (75.6 MHz, CDCl_3_): δ 3.7 (C-CH_3_), 52.8 (CO_2_Me), 55.9 (OMe), 57.4 (O-CH_2_), 73.7 (C-CH_3_), 84.3 (C≡C(CH_3_)), 111.0 (C5′), 115.6 (C2′), 123.3 (CH=C), 125.6 (C6′), 125.9 (aryl C), 126.0 (CH=C), 146.7 (C4′), 150.7 (C3′), 164.2 (CO_2_Me); HRMS (+ESI): Found *m*/*z* 324.0944 [M + Na]^+^, C_15_H_15_N_3_O_4_Na requires 324.0955.

*Methyl (Z)-2-azido-3-(4-(but-2-yn-1-yloxy)-3-methoxyphenyl)acrylate* (**20b**) The *title* compound was prepared as described in GP-2 from 4-(but-2-yn-1-yloxy)-3-methoxybenzaldehyde (19b)(530 mg, 2.6 mmol) and methyl azidoacetate (2.99 g, 26.0 mmol) in anhydrous methanol (30 mL) to give the product (477 mg, 61%) as a pale yellow granular solid; m.p. 94–96 °C; IR (KBr): *ν*_max_ 2920, 2120, 1702, 1592, 1509, 1434, 1377, 1244, 1140, 1011, 801, 763 cm^−1^; UV-vis (CH_3_CN): λ_max_ 324 nm (ε 14,900 cm^−1^ M^−1^); ^1^H-NMR (300 MHz, CDCl_3_): δ 1.87 (t, *J* = 2.3 Hz, 3H, CH_3_), 3.94 (s, 3H, OMe), 3.94 (s, 3H, CO_2_Me), 4.77 (q, *J* = 2.3 Hz, 2H, O-CH2), 6.90 (s, 1H, CH=C), 7.05 (d, *J* = 8.5 Hz, 1H, H5′), 7.38 (dd, *J* = 8.5, 1.8, Hz, 1H, H6′), 7.53 (d, *J* = 1.8 Hz, 1H, H2′); ^13^CNMR (75.6 MHz, CDCl_3_): δ 3.7 (C-CH_3_), 52.8 (CO_2_Me), 55.9 (OMe), 57.1 (O-CH_2_), 73.5 (C-CH_3_), 84.4 (C≡C(CH_3_)), 112.9 (C5′), 113.3 (C2′), 123.4 (aryl C), 124.5 (CH=C), 125.8 (C6′), 126.8 (CH=C), 14.34 (C3′), 149.0 (C4′), 164.2 (CO_2_Me); HRMS (+ESI): Found *m*/*z* 324.0955 [M + Na]^+^, C_15_H_15_N_3_O_4_Na requires 324.0955.

*Methyl (Z)-2-azido-3-(4-methoxy-3-((3-phenylprop-2-yn-1-yl) oxy)phenyl)acrylate* (**25d**) The *title* compound was prepared as described in GP-2 from 4-methoxy-3-((3-phenylprop-2-yn-1-yl)oxy)benzaldehyde (**24d**) (692 mg, 2.6 mmol) and methyl azidoacetate (2.99 g, 26.0 mmol) in anhydrous methanol (30 mL) to give the product (632 mg, 67%) as a pale yellow granular solid; m.p. 118–120 °C; IR (KBr): *ν*_max_ 2921, 2827, 2119, 1674, 1612, 1580, 1506, 1431, 1378, 1275, 1232, 1129, 1008, 966. 873, 809, 753, 686 cm^−1^; UV-vis (CH_3_CN): λ_max_ 234 nm (ε 57,700 cm^−1^ M^−1^), 272 (22,600), 306 (21,800); ^1^H-NMR (300 MHz, CDCl_3_): δ 3.93 (s, 3H, OMe), 3.96 (s, 3H, CO_2_Me), 5.04 (d, *J* = 6.0 Hz, 2H, O-CH2), 6.91 (s, 1H, CH≡C), 6.92 (d, *J* = 8.0 Hz, 1H, H5′), 7.09 (dd, *J* = 8.0, 1.8 Hz, 1H, H6′), 7.27–7.33 (m, 3H, ArH), 7.43–7.46 (m, 2H, ArH), 7.85 (d, *J* = 1.8 Hz, 1H, H2′); ^13^C-NMR (75.6 MHz, CDCl_3_): δ 52.6 (CO_2_Me), 55.9 (OMe), 57.5 (O-CH_2_), 83.9 (C-Ph), 87.5 (C≡C(Ph)), 111.1 (C5′), 116.0 (C2′), 120.4 (aryl C), 125.7 (CH=C), 126.0 (C6′), 126.1 (aryl C), 128.3 (2×aryl CH), 128.6 (CH=C), 130.6 (aryl CH), 131.9 (2×aryl CH), 146.7 (C4′), 150.9 (C3′), 164.2 (CO_2_Me); HRMS (+ESI): Found *m*/*z* 386.1099 [M + Na]^+^, C_20_H_17_N_3_O_4_Na requires 386.1117.

*Methyl (Z)-2-azido-3-(4-methoxy-3-((2-methylbut-3-yn-2-yl) oxy)phenyl)acrylate* (**25c**) The *title* compound was prepared as described in GP-2 from 4-methoxy-3-((2-methylbut-3-yn-2-yl)oxy)benzaldehyde (**24c**) (567 mg, 2.6 mmol) and methyl azidoacetate (2.99 g, 26.0 mmol) in anhydrous methanol (30 mL) to give the product (507 mg, 62%) as a pale yellow granular solid; m.p. 122–124 °C; IR (KBr): *ν*_max_ 2988, 2835, 2094, 1691, 1619, 1565, 1506, 1425, 1374, 1253, 1138, 1084, 1027, 962. 891, 799, 756, 687 cm^−1^; UV-vis (CH_3_CN): λ_max_ 238 nm (ε 16,800 cm^−1^ M^−1^), 328 (35,400); ^1^H-NMR (300 MHz, CDCl_3_): δ 1.70 (s, 6H, 2 × C-CH_3_), 2.59 (d, *J* = 0.9 Hz, 1H, CH≡C), 3.87 (s, 3H, OMe), 3.92 (s, 3H, CO_2_Me), 6.91 (s, 1H, CH=C), 6.90 (d, *J* = 8.5 Hz, 1H, H5′), 7.45 (dd, *J* = 1.9, 8.5 Hz, 1H, H6′), 8.16 (d, *J* = 2.0 Hz, 1H, H2′);^13^C-NMR (75.6 MHz, CDCl_3_): δ 29.3 (2×C-CH_3_), 52.7 (CO_2_Me), 55.7 (OMe), 73.6 (C≡CH), 74.1 (C-(CH_3_)_2_), 86.1 (C≡CH), 111.5 (C5′), 123.2 (C2′), 124.8 (CH=C), 125.7 (C6′), 125.9 (aryl C), 127.4 (CH=C), 144.2 (C4′), 153.9 (C3′), 164.2 (CO_2_Me); HRMS (+ESI): Found *m*/*z* 338.1099 [M + Na]^+^, C_16_H_17_N_3_O_4_Na requires 338.1117.

*Methyl (Z)-2-azido-3-(3-methoxy-4-((2-methylbut-3-yn-2-yl)oxy)phenyl)acrylate* (**20c**) The *title* compound was prepared as described in GP-2 from 3-methoxy-4-((2-methylbut-3-yn-2-yl)oxy)benzaldehyde (**19c**) (567 mg, 2.6 mmol) and methyl azidoacetate (2.1 g, 26. mmol) in anhydrous methanol (30 mL) to give the product (524 mg, 62%) as a pale yellow granular solid; m.p. 116–118 °C; IR (KBr): *ν*_max_ 2975, 2802, 2112, 1703, 1635, 1565, 1512, 1400, 1387, 1212, 1100, 1052, 998, 957, 888, 765, 745, 677 cm^−1^; UV-vis (CH_3_CN): λ_max_ 328 nm (ε 17,100 cm^−1^ M^−1^); ^1^H-NMR (300 MHz, CDCl_3_): δ 1.72 (s, 6H, 2 × C-CH_3_), 2.60 (d, *J* = 0.9 Hz, 1H, CH≡C), 3.89 (s, 3H, OMe), 3.93 (s, 3H, CO_2_Me), 6.91 (s, 1H, CH=C), 7.33 (dd, *J* = 8.5, 1.9 Hz, 1H, H6′), 7.45 (d, *J* = 8.5 Hz, 1H, H5′), 7.52 (d, *J* = 1.9 Hz, 1H, H2′); ^13^C-NMR (75.6 MHz, CDCl_3_): δ 29.4 (2 × C-CH_3_), 52.8 (CO_2_Me), 55.9 (OMe), 73.9 (C≡CH), 73.9 (C-(CH_3_)_2_) 85.8 (C≡CH), 113.9 (C5′), 121.5 (C2′), 123.8 (CH=C), 123.9 (C6′), 125.7 (aryl C), 128.5 (CH=C), 146.1 (C3′), 152.0 (C4′), 164.1 (CO_2_Me); HRMS (+ESI): Found *m*/*z* 338.1104 [M + Na]^+^, C_16_H_17_N_3_O_4_Na requires 338.1117.

*Methyl 6-methoxy-5-(prop-2-yn-1-yloxy)-1H-indole-2-carboxylate* (**26a**) The *title* compound was prepared as described in GP-3 from methyl (*Z*)-2-azido-3-(3-(but-2-yn-1-yloxy)-4-methoxyphenyl)acrylate (**25a**) (596 mg, 2.08 mmol) in xylene (20 mL) to give the product (196 mg, 73%) as a yellow solid; m.p. 164–166 °C; IR (KBr): *ν*_max_ 3325, 3259, 2953, 2925, 2123, 1671, 1624, 1520, 1480, 1440, 1367, 1320, 1249, 1208, 1185, 1146, 1005, 890, 833, 766, 694 cm^−1^; UV-vis (CH_3_CN): λ_max_ 208 nm (ε 40,600 cm^−1^ M^−1^), 318 (28,700); ^1^H-NMR (300 MHz, CDCl_3_): δ 2.55 (t, *J* = 2.4 Hz, 1H, CH≡C), 3.95 (s, 3H, OMe), 3.96 (s, 3H, CO_2_Me), 4.82 (d, *J* = 2.4 Hz, 2H, O-CH_2_), 6.90 (d, *J* = 0.8 Hz, 1H, H4), 7.16 (q, *J* = 0.9 Hz, 1H, H3), 7.28 (s, 1H, H7), 8.91 (bs, 1H, NH), ^13^C-NMR (75.6 MHz, CDCl_3_): δ 51.8 (CO_2_Me), 56.0 (OMe), 57.3 (O-CH_2_), 75.7 (C≡CH), 78.7 (C≡CH), 94.0 (C7), 106.6 (C4), 109.0 (C3), 120.2 (aryl C), 125.9 (C2), 132.9 (aryl C), 143.7 (C6), 150.6 (C5), 162.3 (CO_2_Me); HRMS (+ESI): Found *m*/*z* 282.0725 [M + Na]^+^, C_14_H_13_NO_3_Na requires 282.0742.

*Methyl 5-methoxy-6-(prop-2-yn-1-yloxy)-1H-indole-2-carboxylate* (**21a**) The *title* compound was prepared as described in GP-3 from methyl (*Z*)-2-azido-3-(4-(but-2-yn-1-yloxy)-3-methoxyphenyl)acrylate (**20a**) (596 mg, 2.08 mmol) in xylene (20 mL) to give the product (183 mg, 68%) as a yellow solid; m.p. 152–154 °C; IR (KBr): *ν*_max_ 3325, 3241, 2999, 2937, 2113, 1681, 1638, 1522, 1475, 1452, 1360, 1280, 1237, 1211, 1195, 1143, 1003, 924, 843, 819, 761, 675 cm^−1^; UV-vis (CH_3_CN): λ_max_ 209 nm (ε 35,800 cm^−1^ M^−1^), 308 (21,400); ^1^H-NMR (300 MHz, CDCl_3_): δ 2.57 (t, *J* = 2.4 Hz, 1H, CH≡C), 3.95 (s, 3H, OMe), 3.96 (s, 3H, CO_2_Me), 4.85 (d, *J* = 2.4 Hz, 2H, O-CH_2_), 7.08 (d, *J* = 0.8 Hz, 1H, H4), 7.11 (s, 1H, H7), 7.15 (q, *J* = 0.9 Hz, 1H, H3), 8.87 (bs, 1H, NH); ^13^C-NMR (75.6 MHz, CDCl_3_): δ 51.8 (CO_2_Me), 56.2 (OMe), 56.9 (O-CH_2_), 76.1 (C≡CH), 77.4 (C≡CH), 96.8 (C7), 103.0 (C4), 108.7 (C3), 121.4 (aryl C), 126.1 (C2), 131.6 (aryl C), 146.6 (C5), 147.6 (C6), 162.2 (CO_2_Me); HRMS (+ESI): Found *m*/*z* 282.0726 [M + Na]^+^, C_14_H_13_NO_3_Na requires 282.0742.

*Methyl 5,6-bis(but-2-yn-1-yloxy)-1H-indole-2-carboxylate* (**31**) The *title* compound was prepared as described in GP-3 from methyl (*Z*)-2-azido-3-(3,4-bis(but-2-yn-1-yloxy)phenyl)acrylate (**30**) (704 mg, 2.08 mmol) in xylene (20 mL) to give the product 210 mg, 65%) as a yellow solid; m.p. 146–148 °C; IR (KBr): *ν*_max_ 3332, 2912, 2294, 2120, 1682, 1644, 1519, 1434, 1379, 1244, 1204, 1145, 984, 891, 819, 765 cm^−1^; UV-vis (CH_3_CN): λ_max_ 210 nm (ε 39,600 cm^−1^ M^−1^), 317 (24,900); ^1^H-NMR (300 MHz, CDCl_3_): δ 1.87 (q, *J* = 2.4 Hz, 6H, 2 × C-CH_3_), 3.96 (s, 3H, CO_2_Me), 4.76 (q, *J* = 2.4 Hz, 2H, O-CH2), 4.79 (q, *J* = 2.4 Hz, 2H, O-CH2), 7.08 (d, *J* = 0.7 Hz, 1H, H4), 7.16 (q, *J* = 0.9 Hz, 1H, H3), 7.25 (s, 1H, H7), 9.03 (bs, 1H, NH); ^13^C-NMR (75.6 MHz, CDCl_3_): δ 3.7 (2 × C-CH_3_), 51.8 (CO_2_Me), 57.4 and 57.8 (O-CH_2_), 73.9 (C-CH_3_), 74.2 (C-CH_3_), 83.8 (C≡C(CH_3_)), 84.2 (C≡C(CH_3_)), 96.5 (C7), 106.1 (C4), 108.9 (C3), 120.9 (aryl C), 126.0 (C2), 132.5 (aryl C), 144.4 (C5), 148.5 (C6), 162.3 (CO_2_Me); HRMS (+ESI): Found *m*/*z* 334.1046 [M + Na]^+^, C_18_H_17_NO_4_Na requires 334.1050.

*Methyl 5-(but-2-yn-1-yloxy)-6-methoxy-1H-indole-2-carboxylate* (**26b**) The *title* compound was prepared as described in GP-3 from methyl (*Z*)-2-azido-3-(3-(but-2-yn-1-yloxy)-4-methoxyphenyl)acrylate (**25b**) (626 mg, 2.08 mmol) in xylene (20 mL) to give the product (201 mg, 71%) as a yellow solid; m.p. 160–162 °C; IR (KBr): *ν*_max_ 3324, 3258, 3009, 2918, 2795, 2123, 2108, 1672, 1600, 1520, 1490, 1425, 1400, 1385, 1346, 1249, 1208, 1185, 1146, 1005, 980, 890, 833, 766 cm^−1^; UV-vis (CH_3_CN): λ_max_ 208 nm (ε 27,700 cm^−1^ M^−1^), 319 (18,900);^1^H-NMR (300 MHz, CDCl_3_): δ 1.88 (q, *J* = 2.4 Hz, 3H, C-CH_3_), 3.95 (s, 3H, OMe), 3.96 (s, 3H, CO_2_Me), 4.77 (q, *J* = 2.4 Hz, 2H, O-CH_2_), 6.88 (d, *J* = 0.7 Hz, 1H, H4), 7.16 (q, *J* = 0.9 Hz, 1H, H3), 7.23 (s, 1H, H7), 8.82 (bs, 1H, NH); ^13^C-NMR (75.6 MHz, CDCl_3_): δ 5.2 (C-CH_3_), 53.2 (CO_2_Me), 57.4 (OMe), 59.1 (O-CH_2_), 75.5 (C-CH_3_), 85.3 (C≡C), 95.2 (C7), 107.1 (C4), 110.4 (C3), 121.7 (aryl C), 127.1 (C2), 134.0 (aryl C), 145.5 (C6), 151.9 (C5), 163.7 (CO_2_Me); HRMS (+ESI): Found *m*/*z* 296.0884 [M + Na]^+^, C_15_H_15_NO_4_Na requires 296.0899.

*Methyl 6-(but-2-yn-1-yloxy)-5-methoxy-1H-indole-2-carboxylate* (**21b**) The *title* compound was prepared as described in GP-3 from methyl (*Z*)-2-azido-3-(4-(but-2-yn-1-yloxy)-3-methoxyphenyl)acrylate (**20b**) (626 mg, 2.08 mmol) in xylene (20 mL) to give the product (190 mg, 67%) as a yellow solid; m.p. 164–166 °C; IR (KBr): *ν*_max_ 3324, 3224, 3005, 2936, 2835, 2215, 2108, 1680, 1584, 1495, 1455, 1433, 1362, 1285, 1243, 1228, 1146, 1045, 988, 946, 846, 819, 760, 672 cm^−1^; UV-vis (CH_3_CN): λ_max_ 210 nm (ε 37,100 cm^−1^ M^−1^), 319 (22,300); ^1^H-NMR (300 MHz, CDCl_3_): δ 1.87 (q, *J* = 2.4 Hz, 3H, C-CH_3_), 3.94 (s, 3H, OMe), 3.96 (s, 3H, CO_2_Me), 4.80 (q, *J* = 2.4 Hz, 2H, O-CH2), 7.06 (d, *J* = 0.7 Hz, 1H, H4), 7.08 (s, 1H, H7), 7.14 (q, *J* = 0.9 Hz, 1H, H3), 9.01 (bs, 1H, NH); ^13^C-NMR (75.6 MHz, CDCl_3_): δ 3.7 (C-CH_3_), 51.8 (CO_2_Me), 56.1 (OMe), 57.4 (O-CH_2_), 73.8 (C-CH_3_), 84.3 (C≡C(CH_3_)), 96.2 (C7), 102.7 (C4), 108.7 (C3), 121.0 (aryl C), 125.8 (C2), 131.8 (aryl C), 146.1 (C5), 148.0 (C6), 162.3 (CO_2_Me); HRMS (+ESI): Found *m*/*z* 296.0883 [M + Na]^+^, C_15_H_15_NO_4_Na requires 296.0889.

*Methyl 6-methoxy-5-((3-phenylprop-2-yn-1-yl)oxy)-1H-indole-2-carboxylate* (**26d**) The *title* compound was prepared as described in GP-3 from methyl (Z)-2-azido-3-(4-methoxy-3-((3-phenylprop-2-yn-1-yl)oxy)phenyl)acrylate (**25d**) (754 mg, 2.08 mmol) in xylene (20 mL) to give the product (257 mg, 74%) as a yellow solid; m.p. 138–140 °C; IR (KBr): *ν*_max_ 3326, 2984, 2924, 2740, 2113, 1675, 1645, 1520, 1480, 1439, 1381, 1247, 1195, 1145, 996, 845, 823, 761, 672 cm^−1^; UV-vis (CH_3_CN): λ_max_ 203 nm (ε 58,900 cm^−1^ M^−1^), 318 (22,200); ^1^H-NMR (300 MHz, CDCl_3_): δ 3.95 (s, 3H, OMe), 3.98 (s, 3H, CO_2_Me), 5.04 (s, 2H, O-CH2), 6.90 (s, 1H, H4), 7.17 (q, *J* = 0.9 Hz, 1H, H3), 7.31 (s, 1H, H7), 7.32–7.36 (m, 3H, ArCH), 7.44–7.47 (m, 2H, ArCH), 8.84 (bs, 1H, NH); ^13^C-NMR (75.6 MHz, CDCl_3_): δ 51.8 (CO_2_Me), 56.0 (OMe), 58.2 (O-CH_2_), 84.1 (C≡C(Ph)), 87.4 (C-Ph), 93.9 (C7), 106.6 (C4), 109.0 (C3), 120.3 (aryl C), 122.4 (aryl C), 125.8 (C2), 128.2 (2 × aryl CH), 128.5 (aryl CH), 131.8 (2 × aryl CH), 132.8 (aryl C), 144.0 (C6), 150.7 (C5), 162.2 (CO_2_Me); HRMS (+ESI): Found *m*/*z* 336.1221 [M + H]^+^, C_20_H_18_NO_4_ requires 336.1236.

*Methyl 5,10-dihydro-7H-dipyrano[3,2-e:2′,3′-g]indole-6-carboxylate* (**10**) *Method 2: Route 2* The title compound was prepared as described in GP-3 from methyl (Z)-2-azido-3-(3,4-bis(prop-2-yn-1-yloxy)phenyl) acrylate (**9**) (646 mg, 2.08 mmol) in xylene (20 mL) to give the product (61.4 mg, 72%) as a yellow solid; m.p. 168–170 °C; IR (KBr): ν_max_ 3415, 3328, 2947, 2831, 2341, 2116, 1674, 1640, 1575, 1524, 1486, 1441, 1400, 1274, 1213, 1158, 1135, 1025, 1000, 920, 813, 755 cm^−1^; UV-vis (CH_3_CN): λ_max_ 225 nm (ε 24,100 cm^−1^ M^−1^), 269 (13,800), 360 (13,000); ^1^H-NMR (300 MHz, CDCl_3_): δ 3.96 (s, 3H, CO_2_Me), 4.91 (dd, J = 3.8, 1.8 Hz, 2H, O-CH_2_), 4.94 (dd, *J* = 3.8, 1.8 Hz, 2H, O-CH_2_), 5.87–5.97 (m, 2H, H3 and H9), 6.72–6.80 (m, 2H, H4 and H8), 7.19 (d, *J* = 2.1 Hz, 1H, H5), 8.85 (s, 1H, NH); ^13^C-NMR (75.6 MHz, CDCl_3_): δ52.0 (CO_2_Me), 65.7 (O-CH_2_), 65.8 (O-CH_2_), 106.5 (C5), 107.0 (aryl C), 115.3 (aryl C), 118.8 (C4), 119.3 (C8), 120.8 (aryl C), 121.8 (C3), 121.9 (C9), 126.5 (C6), 128.8 (aryl C), 138.1 (aryl C), 141.7 (aryl C), 162.3 (CO_2_Me); HRMS (+ESI): Found *m*/*z* 306.0736 [M + Na]^+^, C_16_H_13_NO_4_Na requires 306.0737.

This compound was also prepared by the methods described below. Method 1: The title compound was prepared as described in GP-6 from methyl 5,6-bis(prop-2-yn-1-yloxy)-1*H*-indole-2-carboxylate (**16**) (118 mg, 0.42 mmol) in chlorobenzene (30 mL) to give the product (43.5 mg, 51%) as a yellow solid.

Method 2: Route 1 The title compound was prepared as described in GP-6 from methyl (*Z*)-2-azido-3-(2*H*,10*H*-pyrano[4,3-h]chromen-5-yl)acrylate (**17**) (646 mg, 2.08 mmol) in xylene (20 mL) to give the product (52.9 mg, 62%) as a yellow solid.

*Methyl 5-methoxy-3,7-dihydropyrano[3,2-e]indole-2-carboxylate* (**27a**) The title compound was prepared as described in GP-6 from methyl 6-methoxy-5-(prop-2-yn-1-yloxy)-1H-indole-2-carboxylate (**26a**) (108 mg, 0.42 mmol) in chlorobenzene (30 mL) to give the product (80 mg, 74%) as a yellow solid; m.p. 166–168 °C; IR (KBr): ν_max_ 3325, 2948, 2839, 2340, 2110, 1677, 1637, 1515, 1439, 1271, 1193, 1143, 1098, 998, 931, 883, 808, 752 cm^−1^; UV-vis (CH_3_CN): λ_max_ 212 nm (ε 37,400 cm^−1^ M^−1^), 271 (13,300), 322 (30,600); ^1^H-NMR (300 MHz, CDCl_3_): δ 3.92 (s, 3H, OMe), 3.93 (s, 3H, CO_2_Me), 4.90 (dd, *J* = 3.7, 1.7 Hz, 2H, O-CH2), 5.89–5.95 (m, 1H, H8), 6.76–6.79 (m, 1H, H9), 6.79 (s, 1H, H4), 7.17 (d, *J* = 2.1 Hz, 1H, H1), 8.96 (s, 1H, NH); ^13^C-NMR (75.6 MHz, CDCl_3_): δ 51.8 (CO_2_Me), 56.0 (OMe), 65.7 (O-CH_2_), 93.8 (C4), 106.1 (C1), 114.9 (aryl C), 118.3 (aryl C), 121.6 (C9), 121.8 (C8), 126.0 (C2), 132.2 (aryl C), 139.3 (aryl C), 148.8 (C5), 162.2 (CO_2_Me); HRMS (+ESI): Found *m*/*z* 282.0731 [M + Na]^+^, C_14_H_13_NO_4_Na requires 282.0737.

*Methyl 5-methoxy-1,7-dihydropyrano[2,3-g]indole-2-carboxylate* (**22a**) The *title* compound was prepared as described in GP-6 from methyl 5-methoxy-6-(prop-2-yn-1-yloxy)-1*H*-indole-2-carboxylate (**21a**) (108 mg, 0.42 mmol) in chlorobenzene (30 mL) to give the product (83 mg, 77%) as a yellow solid; m.p. 188–190 °C; IR (KBr): *ν*_max_ 3327, 2944, 2827, 2366, 2106, 1677, 1528, 1444, 1306, 1221, 1148, 1101, 989, 955, 831, 755 cm^−1^; UV-vis (CH_3_CN): λ_max_ 226 nm (ε 28,100 cm^−1^ M^−1^), 290 (16,700), 339 (18,400); ^1^H-NMR (300 MHz, CDCl_3_): δ 3.94 (s, 3H, OMe), 3.96 (s, 3H, CO_2_Me), 4.96 (dd, *J* = 3.8, 1.8 Hz, 2H, O CH_2_), 5.89–5.95 (m, 1H, H8), 6.75–6.79 (m, 1H, H9), 7.02 (s, 1H, H4), 7.13 (d, *J* = 2.1 Hz, 1H, H3), 8.94 (s, 1H NH); ^13^C-NMR (75.6 MHz, CDCl_3_): δ51.9 (CO_2_Me), 56.2 (OMe), 65.7 (O-CH_2_), 103.1 (C4), 107.1 (aryl C), 109.2 (C3), 119.3 (aryl C), 120.8 (C8), 121.0 (C9), 126.0 (C2), 128.7 (aryl C), 137.6 (aryl C), 145.2 (C5), 162.3 (CO_2_Me); HRMS (+ESI): Found *m*/*z* 282.0738 [M + Na]^+^, C_14_H_13_NO_4_Na requires 282.0737.

*Methyl 5-methoxy-9-methyl-3,7-dihydropyrano[3,2-e]indole-2-carboxylate* (**27b**) The *title* compound was prepared as described in GP-6 from methyl 5-(but-2-yn-1-yloxy)-6-methoxy-1*H*-indole-2-carboxylate (**26b**) (114 mg, 0.42 mmol) in chlorobenzene (30 mL) to give the product (62 mg, 54%) as a yellow solid; m.p. 196–198 °C; IR (KBr): *ν*_max_ 3310, 2948, 2925, 2833, 2106, 1675, 1640, 1517, 1495, 1439, 1366, 1274, 1265, 1194, 1146, 1080, 1004, 960, 880, 815, 765, 672 cm^−1^; UV-vis (CH_3_CN): λ_max_ 213 nm (ε 28,900 cm^−1^ M^−1^), 267 (9,500), 322 (24,200); ^1^H-NMR (300 MHz, CDCl_3_): δ 1.63 (s, 3H, C-CH_3_), 3.94 (s, 3H, OMe), 3.95 (s, 3H, CO_2_Me), 4.77 (q, *J* = 2.4 Hz, 2H, O-CH_2_), 5.69–5.72 (m, 1H, H8), 6.87 (s, 1H, H1), 7.23 (s, 1H, H4), 8.82 (bs, 1H, NH); ^13^C-NMR (75.6 MHz, CDCl_3_): δ 22.6 (C-CH_3_), 51.8 (CO_2_Me), 56.1 (OMe), 65.2 (O-CH_2_), 93.8 (C4), 109.3 (C1), 117.4 (aryl C), 117.9 (aryl C), 118.3 (C8), 126.5 (C2), 132.0 (C-CH_3_), 132.9 (aryl C), 140.4 (aryl C), 149.0 (C5), 162.2 (CO_2_Me); HRMS (+ESI): Found *m*/*z* 296.0887 [M + Na]^+^, C_15_H_15_NO_4_Na requires 296.0893.

*Methyl 5-methoxy-9-methyl-1,7-dihydropyrano[2,3-g]indole-2-carboxylate* (**22b**) The *title* compound was prepared as described in GP-6 from methyl 6-(but-2-yn-1-yloxy)-5-methoxy-1*H*-indole-2-carboxylate (**21b**) (114 mg, 0.42 mmol) in chlorobenzene (30 mL) to give the product (56 mg, 49%) as a yellow solid; m.p. 176–178 °C; IR (KBr): *ν*_max_ 3410, 2980, 2930, 2845, 1695, 1645, 1606, 1534, 1445, 1430, 1385, 1375, 1232, 1189, 1139, 1096, 1040, 998, 981, 927, 857, 744, 712cm^−1^; UV-vis (CH_3_CN): λ_max_ 224 nm (ε 32,500 cm^−1^ M^−1^), 286 (22,200), 337 (23,500); ^1^H-NMR (300 MHz, CDCl_3_): δ 1.71 (s, 3H, C-CH_3_), 3.94 (s, 3H, OMe), 3.96 (s, 3H, CO_2_Me), 4.77 (q, *J* = 1.6 Hz, 2H, O-CH_2_), 5.65–5.68 (m, 1H, H8), 7.05 (s, 1H, H4), 7.14 (d, *J* = 2.1 Hz, 1H, H3), 8.81 (bs, 1H, NH), ^13^C-NMR (75.6 MHz, CDCl_3_): δ 20.8 (C-CH_3_), 51.8 (CO_2_Me), 56.2 (OMe), 65.4 (O-CH_2_), 103.2 (C4), 108.9 (C3), 109.9 (aryl C), 117.7 (C8), 121.9 (aryl C), 126.0 (C2), 128.7 (C-CH_3_), 129.3 (aryl C), 144.2 (aryl C), 145.4 (C5), 162.1 (CO_2_Me); HRMS (+ESI): Found *m*/*z* 296.0886 [M + Na]^+^, C_15_H_15_NO_4_Na requires 296.0893.

*Methyl 5-methoxy-9-phenyl-3,7-dihydropyrano[3,2-e]indole-2-carboxylate* (**27d**) The *title* compound was prepared as described in GP-6 from methyl 6-methoxy-5-((3-phenylprop-2-yn-1-yl)oxy)-1*H*-indole-2-carboxylate (**26d**) (140 mg, 0.42 mmol) in chlorobenzene (30 mL) to give the product (113 mg, 81%) as a yellow solid; m.p. 188–190 °C; IR (KBr): *ν*_max_ 3311, 2949, 2816, 2114, 1684, 1639, 1598, 1515, 1500, 1438, 1400, 1360, 1260, 1241, 1193, 1141, 1042, 1011, 995, 920, 823, 759, 703 cm^−1^; UV-vis (CH_3_CN): λ_max_ 274 nm (ε 11,600 cm^−1^ M^−1^), 326 (26,400); ^1^H-NMR (300 MHz, CDCl_3_): δ 3.82 (s, 3H, OMe), 4.00 (s, 3H, CO_2_Me), 4.85 (d, *J* = 4.4 Hz, 2H, O-CH_2_), 5.94–5.97 (m, 1H, H8), 5.98 (s, 1H, H4), 6.88 (s, 1H, H1), 7.30–7.34 (m, 2H, ArH), 7.41–7.44 (m, 3H, ArH), 8.76 (bs, 1H, NH), ^13^C-NMR (75.6 MHz, CDCl_3_): δ 51.7 (CO_2_Me), 58.1 (OMe), 65.1 (O-CH_2_), 94.2 (C4), 109.4 (C1), 117.0 (aryl C), 117.5 (aryl C), 125.0 (C2), 120.4 (C8), 127.9 (aryl CH), 128.3 (2 × arylCH), 128.5 (2 × aryl CH), 132.8 (aryl C), 139.4 (C-Ph), 139.4 (aryl C), 141.1 (aryl C), 149.0 (C5), 162.1 (CO_2_Me); HRMS (+ESI): Found *m*/*z* 358.1057 [M + Na]^+^, C_20_H_17_NO_4_Na requires 358.1055.

*Methyl 5-methoxy-7,7-dimethyl-3,7-dihydropyrano[3,2-e] indole-2-carboxylate* (**27c**) The title compound was prepared as described in GP-3 from methyl (*Z*)-2-azido-3-(4-methoxy-3-((2-methylbut-3-yn-2-yl)oxy)phenyl)acrylate (**25c**) (654 mg, 2.08 mmol) in xylene (20 mL) to give the product (220 mg, 68%) as a yellow solid; m.p. 144–146 °C; IR (KBr): *ν*_max_ 3316, 2962, 2975, 2111, 1681, 1625, 1600, 1510, 1439, 1400, 1366, 1270, 1230, 1192, 1131, 1075, 999, 934, 887, 818, 754, 728 cm^−1^; UV-vis (CH_3_CN): λ_max_ 219 nm (ε 40,000 cm^−1^ M^−1^), 271 (13,500), 323 (31,100); ^1^H-NMR (300 MHz, CDCl_3_): δ 1.53 (s, 6H, 2 × CH_3_), 3.92 (s, 3H, OMe), 3.95 (s, 3H, CO_2_Me), 5.73 (d, *J* = 9.7 Hz, 1H, H8), 6.68 (d, *J* = 9.7 Hz, 1H, H9), 6.79 (s, 1H, H4), 7.19 (d, *J* = 2.1 Hz, 1H, H1), 9.00 (bs, 1H, NH); ^13^C-NMR (75.6 MHz, CDCl_3_): δ 27.3 (2 × C-CH_3_), 51.7 (CO_2_Me), 58.1 (OMe), 76.2 (C-(CH_3_)_2_), 93.1 (C4), 106.1 (C1), 113.4 (aryl C), 118.2 (aryl C), 119.3 (C9), 125.7 (C2), 130.6 (C8), 132.0 (aryl C), 138.1 (aryl C), 149.5 (C5), 162.3 (CO_2_Me); HRMS (+ESI): Found *m*/*z* 310.1044 [M + Na]^+^, C_16_H_17_NO_4_Na requires 310.1050.

*Methyl 5-methoxy-7,7-dimethyl-1,7-dihydropyrano[2,3-g] indole-2-carboxylate* (**22c**) The title compound was prepared as described in GP-3 from methyl (*Z*)-2-azido-3-(3-methoxy-4-((2-methylbut-3-yn-2-yl)oxy)phenyl)acrylate (**20c**) (654 mg, 2.08 mmol) in xylene (20 mL) to give the product (203 mg, 74%) as a yellow solid; m.p. 158–160 °C; IR (KBr): ν_max_ 3338, 2955, 2919, 2845, 2111, 1676, 1640, 1595, 1534, 1444, 1370, 1350, 1310, 1265, 1217, 1195, 1134, 1099, 1050, 985, 960, 880, 826, 760, 714 cm^−1^; UV-vis (CH_3_CN): λ_max_ 226 nm (ε 29,600 cm^−1^ M^−1^), 262 (15,200), 341 (19,000); ^1^H-NMR (300 MHz, CDCl_3_): δ 1.55 (s, 6H, 2 × CH_3_), 3.93 (s, 3H, OMe), 3.96 (s, 3H, CO_2_Me), 5.71 (d, *J* = 9.7 Hz, 1H, H8), 6.66 (d, *J* = 9.7 Hz, 1H, H9), 7.02 (s, 1H, H4), 7.13 (d, *J* = 2.1 Hz, 1H, H3), 8.95 (bs, 1H, NH); ^13^C-NMR (75.6 MHz, CDCl_3_): δ 27.4 (2 × C-CH_3_), 51.8 (CO_2_Me), 56.5 (OMe), 76.6 (C-(CH_3_)_2_), 103.3 (C4), 105.8 (aryl C), 109.3 (C3), 116.8 (C9), 120.6 (aryl C), 125.7 (C2), 128.9 (aryl C), 129.8 (C8), 142.3 (aryl C), 149.9 (C5), 162.4 (CO_2_Me); HRMS (+ESI): Found *m*/*z* 288.1228 [M + H]^+^, C_16_H_18_NO_4_ requires 288.1236.

### 3.2. Cell Biology Techniques

The SH-SY5Y and Kelly human neuroblastoma cell lines were generously donated by Dr. J. Biedler (Memorial Sloan-Kettering Cancer Center, New York, NY, USA). The MDA-MB-231 and MCF-7 breast cancer cell lines were purchased from the American Type Culture Collection. All cell lines were cultured under standard conditions at 37 °C in 5% CO_2_ as an adherent monolayer in Dulbecco’s modified Eagle’s medium supplemented with l-glutamine (DMEM) (Invitrogen, Waltham, MA, USA) and 10% fetal calf serum (FCS) (Thermo Fisher Scientific, Waltham, MA, USA).

#### Method for Cell Viability Assays

Cell viability was measured by the standard Alamar blue assay, as previously described [18]. Briefly, cells were allowed to attach for 24 h in 96-well culture plates. The cells were then continuously exposed to serial dilutions of the hydrazide-hydrazone derivatives for 72 h, either in the presence or absence of SAHA (0.5 or 1 µM), with five replicate wells for each determination. Cell viability was determined by the addition of 22 µL of Alamar blue reagent, recorded at comparative 0 h and 5 h values, using a Wallac 1420 Victor III spectrophotometer (GMI, Ramsey, MN, USA), which measured light absorbance in each well at 570 nm. The cell viability of each plate was calculated as a percentage compared to matched DMSO controls (0.5%). The mean (+/’SEM) is shown for three independent experiments.

### 3.3. Statistical Analysis

Results of the cell viability studies were statistically analyzed using the two-tailed, unpaired Student’s t-test. Results are expressed as mean values with 95% confidence intervals.

## 4. Conclusions

The studies presented in this study have contributed to the investigation of the potential of dihydropyranoindoles to act as SAHA enhancers for the treatment of neuroblastoma and breast cancer cells. To the best of our knowledge, this is the first study which was carried out on the combination of SAHA with dihydropyranoindoles. The desired tricyclic and tetracyclic dihydropyranoindoles was achieved by the reaction of corresponding hydroxybenzaldehydes with haloalkynes followed by the application of the Hemetsberger indole synthesis to yield the related indoles. Claisen cyclization in the presence of chlorobenzene afforded the desired compounds in good yields. Some of the dihydropyranoindoles were successfully synthesized from the corresponding azido intermediates in one step by the thermal decomposition step of the Hemetsberger indole synthesis. The biological studies revealed that the breast cancer cells displayed significant resistance towards the combination treatments of the dihydropyranoindoles compared to the neurobalstoma cells. It was also found that tetracyclic analogues of dihydropyranoindoles **33** and **10** were found to be more favorable for the enhancement of SAHA activity, while only dihydropyrano[3,2-*e*]indole tricyclic systems **27a** and **27c** resulted in the additional reduction of the SAHA cytotoxicity against the neuroblastoma cell lines. Taken altogether, the tetracyclic dihydropyranoindole **10** was determined as the most cytotoxic compound at the concentration 20 μM, while the lower concentration of the designated compound displayed the best enhancement on SAHA activity with the value of 44% additional reduction at the ratio of 1:10 SAHA to compound.

## Figures and Tables

**Table:** 

Compound	R_1_	R_2_	R_3_
**27a**	H	H	H
**27b**	H	H	Me
**27c**	Me	Me	H
**27d**	H	H	Ph

## Supplementary Materials

The following figures are available online: **Figure S1** Cell viability of A) SH-SY5Y, B) MDA-MB-231 cancer cells treated with the selected six compounds **1**–**6** (10 μM) over 72 h. Error bars represent mean values (±S.D.) for three independent determinations. **Figure S2**. Comparative toxicity of compounds **1, 3** and **4** (10 µM) against SH-SY5Y, MDA-MB-231 and MRC-5 cell lines after 72 h exposure. Error bars represent mean values (±S.D.) for three independent determinations. **Figure S3**. Cell viability of A) MCF-7 *p* < 0.01 B) MDA-MB-231 *p* < 0.05 breast cancer cells treated with 10 μM compounds over 72 h, in the presence or absence of 1 μM of SAHA. Error bars represent mean values (±S.D.) for three independent determinations **Figure S4**. Cell viability of A) Kelly *p* ≥ 0.05 (ns) B) SH-SY5Y *p* < 0.0005 neuroblastoma cancer cellstreated with 10 μM compounds over 72 h, in the presence or absence of 1 μM of SAHA. Error bars represent mean values (±S.D.) for three independent determinations. **Figure S5**. Cell viability of KELLY neuroblastoma cancer cells treated with compounds A) **33** *p* < 0.01 B) **27c** *p* < 0.05 and C) **10** *p* < 0.05 at different concentrations (0.01, 0.1, 1, 10, 20 μM) over 72 h in the absence and presence of SAHA. Error bars represent mean values (±S.D.) for three independent determinations. **Figure S6**. Cell viability of SH-SY5Y neuroblastoma cancer cells treated with compounds A) **27a**, *p* < 0.0005 B) **33**, *p* < 0.0005 C) **10**, *p* < 0.01 and D) **27c**, *p* < 0.01 at different concentrations (0.01, 0.1, 1, 10, 20 μM) over 72 h in the absence and presence of SAHA. Error bars represent mean values (±S.D.) for three independent determinations. **Figure S7**. Comparative toxicity of compounds A) **27a**, *p* < 0.05 B) **33**, *p* < 0.05 C) **10**, *p* < 0.05 and D) **27c**, *p* < 0.0005 (10 µM) against cancer cells (Kelly, SH-SY5Y, MCF-7 and MDA-MB-231) and human lung fibroblasts (WI-38 and MRC-5) cell lines after 72 h exposure. Error bars represent mean values (±S.D.) for three independent determinations.
