# Supplementary material for: Synthesis, Characterization and Biological Evaluation of Novel Dihydropyranoindoles Improving the Anticancer Effects of HDAC Inhibitors
Source: Molecules. 2020 Mar 18;25(6):1377. doi: 10.3390/molecules25061377 (PMC7144403; doi:10.3390/molecules25061377)
Supplement: Supplementary file 1 [file molecules-25-01377-s001.pdf]

# Synthesis, Characterization and Biological Evaluation of Novel Dihydropyranoindoles Improving the Anticancer Effects of HDAC Inhibitors


**Murat Bingul[1,2,3], Greg M. Arndt[2,4], Glenn M. Marshall[2,3,5], Belamy B. Cheung[2*], Naresh Kumar[1*] and David StC. Black[1*]**

[1] School of Chemistry, UNSW Sydney, Sydney, NSW 2052, Australia; muratbingul1983@gmail.com

[2] Children's Cancer Institute, Lowy Cancer Research Centre, UNSW Sydney, Sydney, NSW 2052, Australia; garndt@ccia.unsw.edu.au (G.M.A.); gmarshall@ccia.unsw.edu.au (G.M.M.)

[3] School of Pharmacy, Dicle University, Diyarbakır, 21280, Turkey

[4] ACRF Drug Discovery Centre for Childhood Cancer, Children's Cancer Institute, Lowy Cancer Research Centre, UNSW Sydney, Sydney, NSW 2052, Australia

[5] Kids Cancer Centre, Sydney Children's Hospital, Randwick, NSW 2031, Australia

[6] School of Women's and Children's Health, UNSW Sydney, Sydney, NSW 2052, Australia

* Correspondence: bcheung@ccia.unsw.edu.au (B.B.C.); n.kumar@unsw.edu.au (N.K.); d.black@unsw.edu.au (D.StC.B.); Tel.: +61-2-9385-2450 (B.B.C.); +61-2-9385-4698 (N.K.); +61-2-9385-4657 (D.StC.B.)

A)

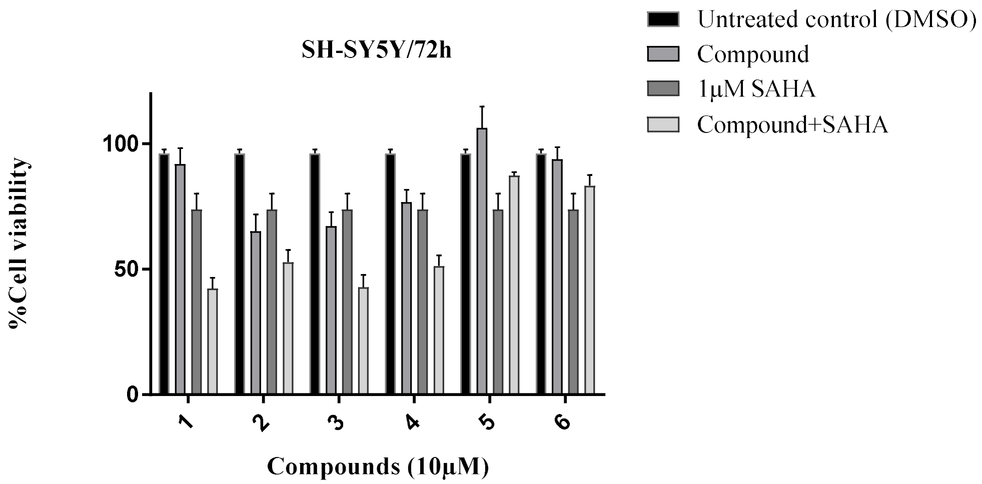


B)

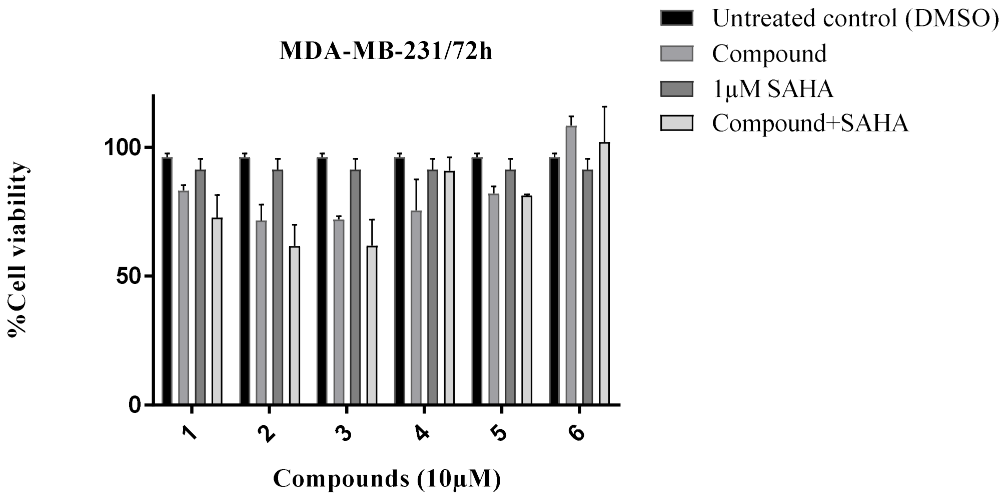


**Figure S1**. Cell viability of A) SH-SY5Y, B) MDA-MB-231 cancer cells treated with the selected six compounds **1-6** (10 µM) over 72 h. Error bars represent mean values (± S.D.) for three independent determinations.

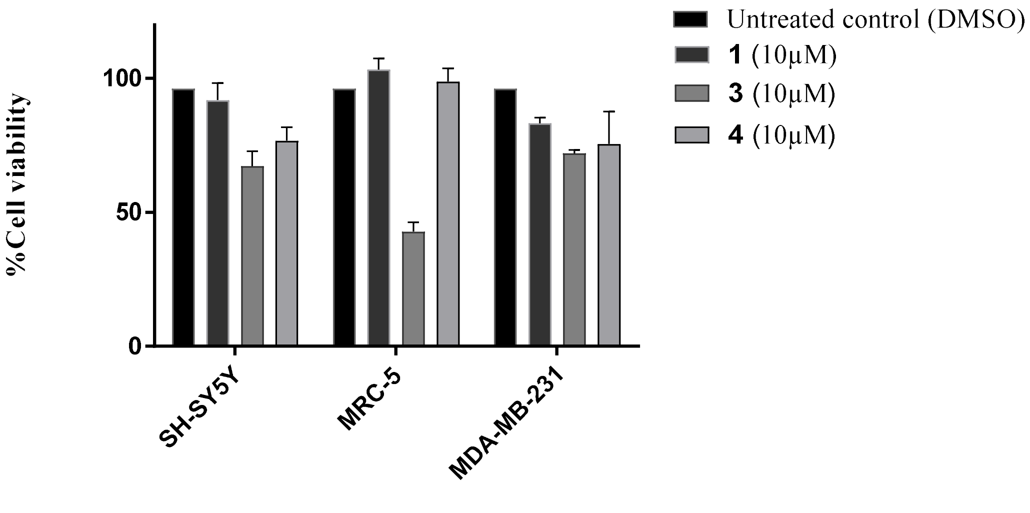


**Figure S2**. Comparative toxicity of compounds **1, 3** and **4** (10 μM) against SH-SY5Y, MDA-MB-231 and MRC-5 cell lines after 72 h exposure. Error bars represent mean values (± S.D.) for three independent determinations.

A)

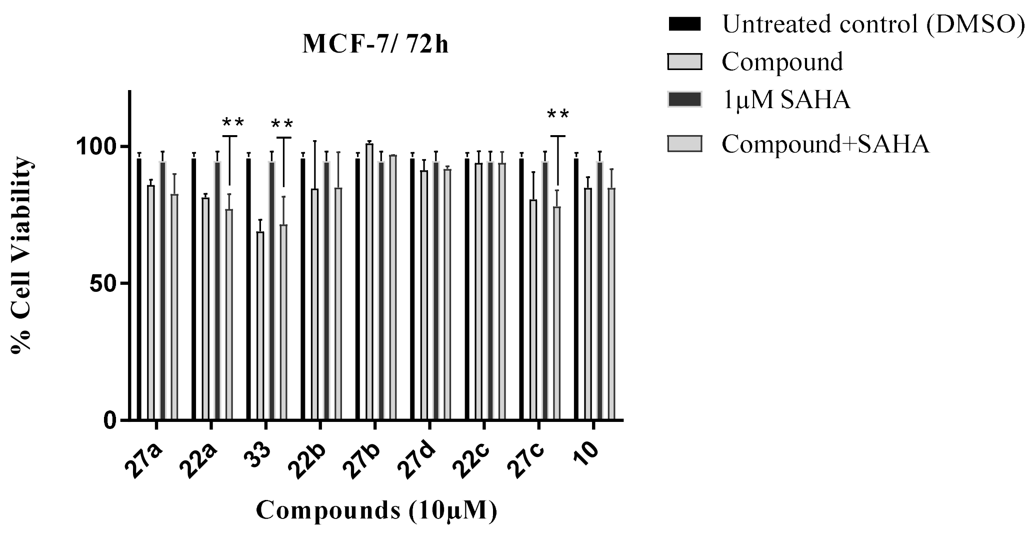


B)

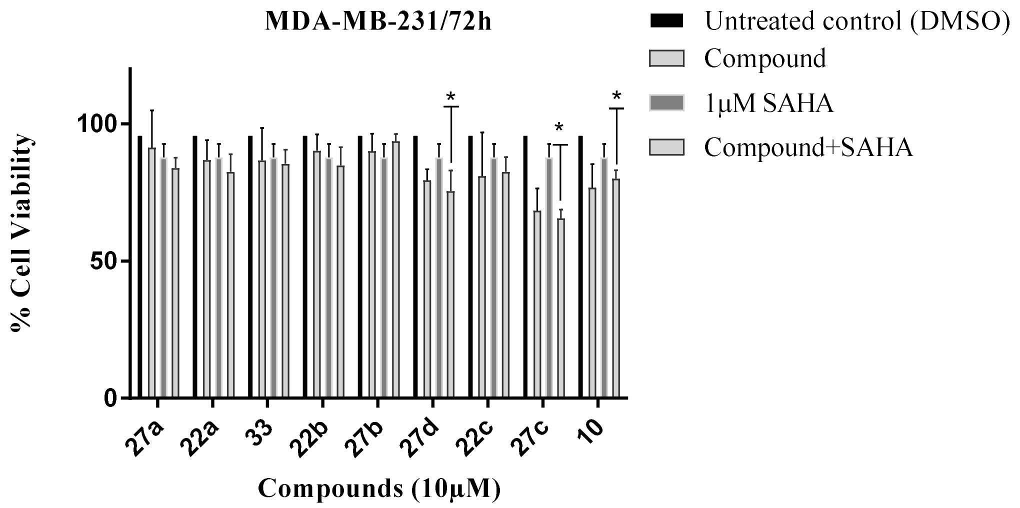


**Figure S3**. Cell viability of A) MCF-7 $p< 0.01$ B) MDA-MB-231 $p< 0.05$ breast cancer cells treated with 10 μM compounds over 72 h, in the presence or absence of 1 μM of SAHA. Error bars represent mean values (± S.D.) for three independent determinations

A)

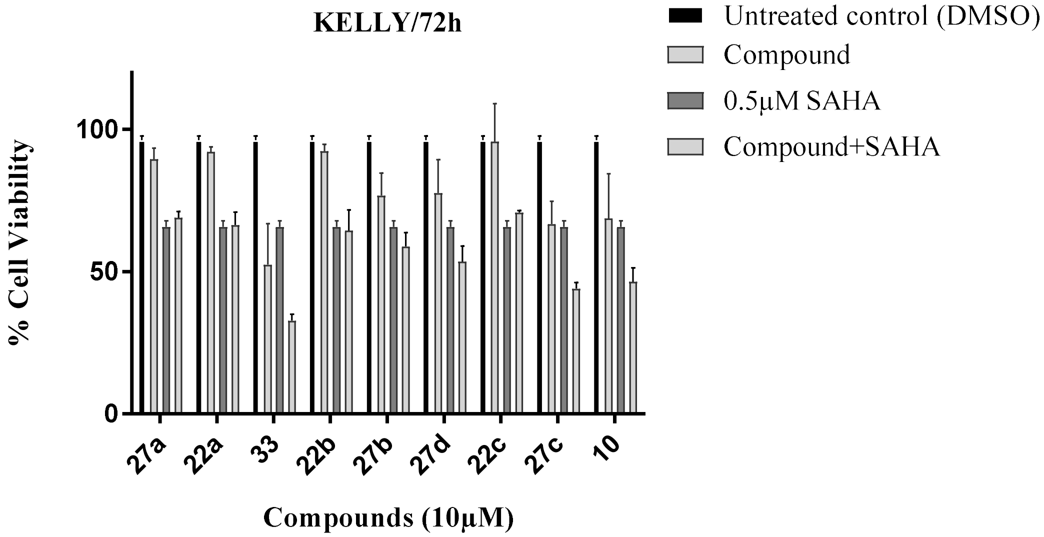


B)

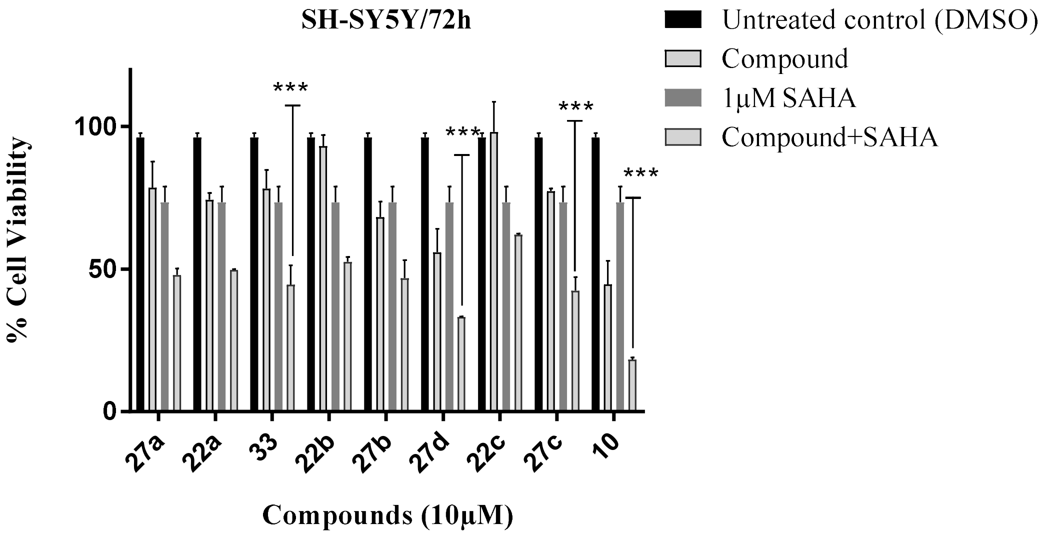


**Figure S4**. Cell viability of A) Kelly $p \geq 0.05$ (ns) B) SH-SY5Y $p < 0.0005$ neuroblastoma cancer cellstreated with 10 µM compounds over 72 h, in the presence or absence of 1 µM of SAHA. Error bars represent mean values (± S.D.) for three independent determinations

A)

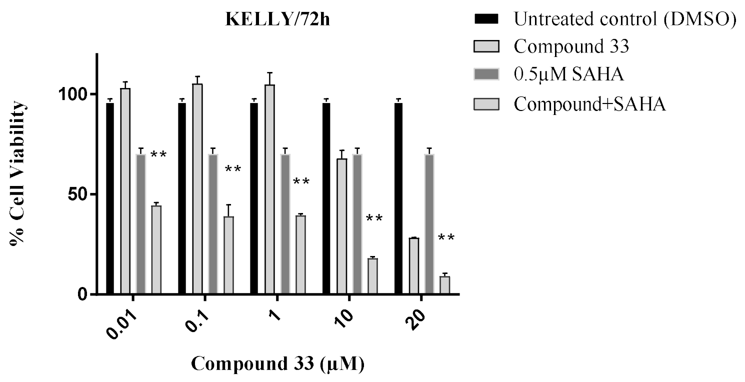


B)

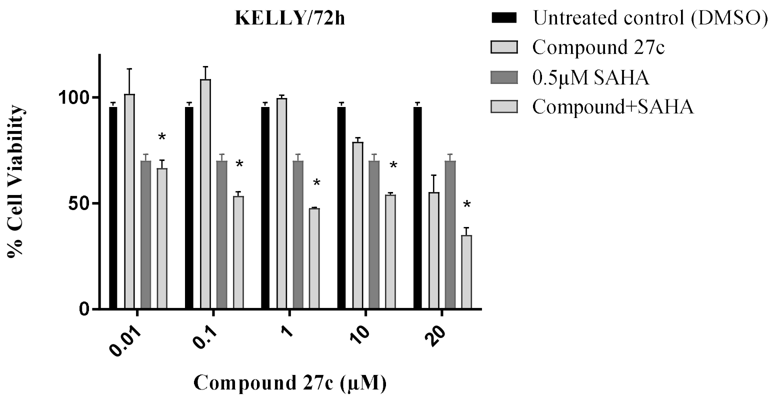


C)

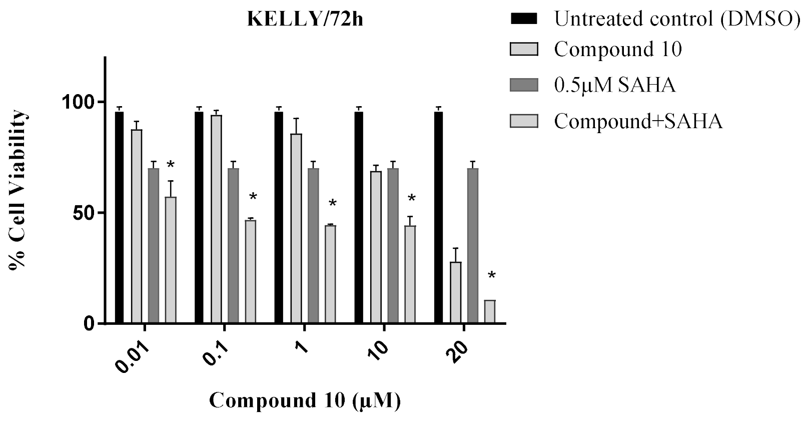


**Figure S5**.Cell viability of KELLY neuroblastoma cancer cells treated with compounds A) **33** $p < 0.01$ B) **27c** $p < 0.05$ and C) **10** $p < 0.05$ at different concentrations (0.01, 0.1, 1, 10, 20 μM) over 72 h in the absence and presence of SAHA. Error bars represent mean values (± S.D.) for three independent determinations.

A)

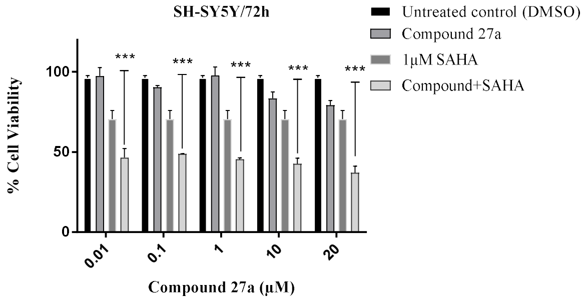


B)

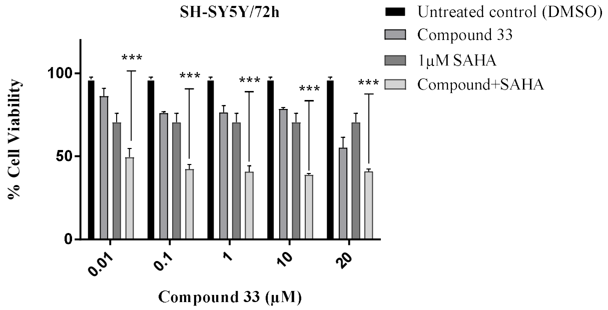


C)

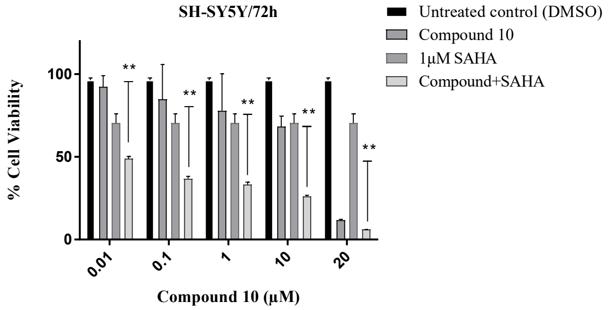


D)

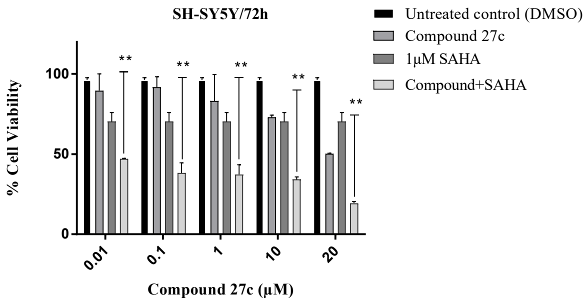


**Figure S6**. Cell viability of SH-SY5Y neuroblastoma cancer cells treated with compounds A) **27a**, $p < 0.0005$ B) **33**, $p < 0.0005$ C) **10**, $p < 0.01$ and D) **27c**, $p < 0.01$ at different concentrations (0.01, 0.1, 1, 10, 20 μM) over 72 h in the absence and presence of SAHA. Error bars represent mean values (± S.D.) for three independent determinations.

A)

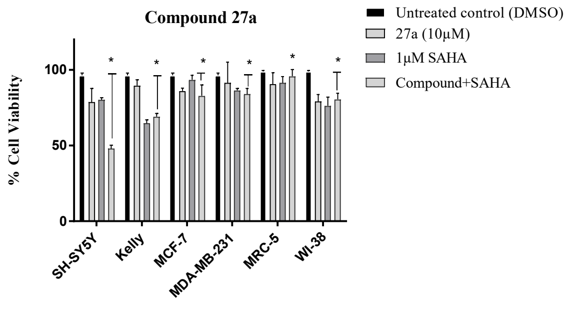


B)

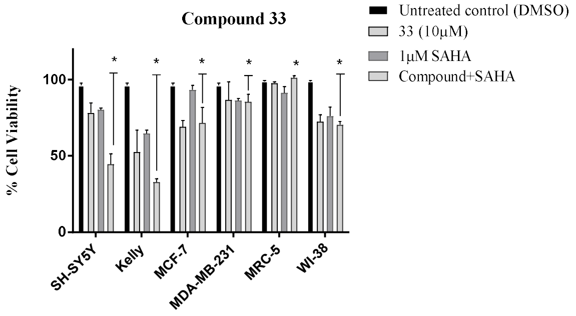


C)

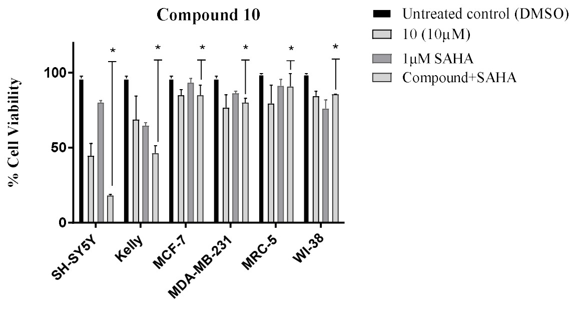


D)

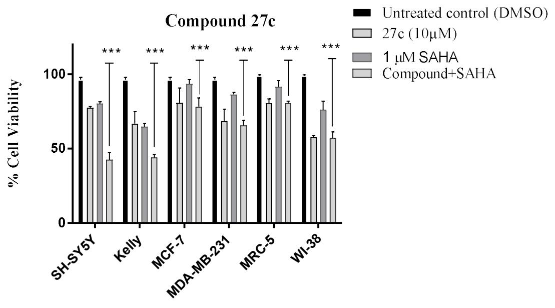


**Figure S7**. Comparative toxicity of compounds A) **27a**, $p< 0.05$ B) **33**, $p< 0.05$ C) **10**, $p< 0.05$ and D) **27c**, $p< 0.0005$ (10 μM) against cancer cells (Kelly, SH-SY5Y, MCF-7 and MDA-MB-231) and human lung fibroblasts (WI-38 and MRC-5) cell lines after 72 h exposure. Error bars represent mean values (± S.D.) for three independent determinations.
